# De-regulated STAT5A/miR-202-5p/USP15/Caspase-6 regulatory axis suppresses CML cell apoptosis and contributes to Imatinib resistance

**DOI:** 10.1186/s13046-019-1502-7

**Published:** 2020-01-17

**Authors:** Zi-Yuan Nie, Min Yao, Zhan Yang, Lin Yang, Xiao-Jun Liu, Jing Yu, Ying Ma, Nan Zhang, Xiao-Yan Zhang, Meng-Han Liu, Ling-Ling Jiang, Jian-Min Luo

**Affiliations:** 1 Department of Hematology, The Second Hospital of Hebei Medical University, 215 Heping W Rd, Shijiazhuang, 050000 China; 2 Department of Biochemistry and Molecular Biology, The Key Laboratory of Neural and Vascular Biology, Ministry of Education of China, Hebei Medical University, No. 361 Zhongshan E Rd, Shijiazhuang, 050017 China; 3 Department of Urology, The Second Hospital of Hebei Medical University, 215 Heping W Rd, Shijiazhuang, 050000 China

**Keywords:** CML, USP15, STAT5A, miRNA, Caspase-6

## Abstract

**Background:**

STAT5 plays an important role in the transformation of hematopoietic cells by BCR-ABL. However, the downstream target genes activated by STAT5 in chronic myeloid leukemia (CML) cells remain largely unclear. Here, we investigated the mechanistic functional relationship between STAT5A-regulated microRNA and CML cell apoptosis.

**Methods:**

The expression of USP15, Caspase-6, STAT5A-regulated miR-202-5p and STAT5A was detected by qRT-PCR and Western blotting in CML cell lines and PBMCs of CML patients. Cell apoptosis was evaluated by flow cytometry. Both gain- and loss-of-function experiments were used to investigate the roles of USP15, miR-202-5p and STAT5A in CML. Luciferase reporter assay detected the effect of miR-202-5p on USP15 expression. Xenograft animal model was used to test the effect of anti-miR-202-5p and pimozide on K562 cell xenograft growth.

**Results:**

USP15 expression was significantly downregulated in CML cell lines and PBMCs of CML patients. Depletion of USP15 increased, whereas overexpression of USP15 reduced the resistance of CML cells to Imatinib. Further, decreased deubiquitinating activity of USP15 by USP15 downregulation led to reduced caspase-6 level, thus attenuating CML cell apoptosis. Mechanistically, miR-202-5p was upregulated in K562G cells and negatively regulated USP15 expression by directly targeting USP15 3′-UTR. Correspondingly, upregulation of miR-202-5p enhanced the resistance of CML cells to Imatinib by inhibiting cell apoptosis. Importantly, STAT5A was upregulated in CML cells and directly activated miR-202-5p transcription by binding to the pre-miR-202 promoter. Pimozide induced CML cell apoptosis and significantly reduced K562 cell xenograft growth in vivo by blocking STAT5A/miR-202-5p/USP15/Caspase-6 regulatory axis.

**Conclusions:**

we provide the first evidence that de-regulated STAT5A/miR-202-5p/USP15/Caspase-6 regulatory axis suppresses the apoptosis of CML cells, targeting this pathway might be a promising therapeutic approach for the treatment of CML.

## Background

Chronic myeloid leukemia (CML) is a clonal disease of pluripotent hematopoietic cells characterized by the expression of the BCR/ABL1 fusion gene, which encodes a constitutively active tyrosine kinase BCR-ABL [1, 2]. The elevated activity of BCR-ABL tyrosine kinase initiates CML and approximately 30% of acute lymphoblastic leukemia (ALL) by stimulating proliferation signals, such as Ras, phosphoinositide 3-kinase (PI3K)/protein kinase B (AKT) and signal transducer and activator of transcriptions (STATs) as well as by inhibiting apoptosis signals, such as Ras-dependent signaling pathway [3, 4].

The deubiquitinating enzyme ubiquitin-specific peptidase 15 (USP15) regulates diverse cellular processes in cancer [5]. USP15 has been shown to stabilize TGF-β receptor I and promote oncogenesis through the activation of TGF-β signaling in glioblastoma [6], and the USP15 gene is also amplified myeloma and ovarian cancers [7, 8]. Currently, the identified targets for USP15 include numerous cancer-associated proteins, such as pro-apoptotic caspase-3 [9], the transforming growth factor beta receptor [6], p53 [5], human homolog of mouse double minute 2 (MDM2) [10], and β-catenin [11]. However, the mechanistic and functional links among STAT5-regulated microRNA, USP15 and target protein of USP15 in CML cells remain poorly understood.

The phosphorylation of STAT5A is essential for the transformation of hematopoietic cells by BCR-ABL and plays an important role in resistance to apoptosis through enhancing the expression of anti-apoptotic factor Bcl-XL [4]. BCR-ABL has been shown to directly induce a tyrosine-phosphorylation and dimerization of STAT5 followed by nuclear translocation of the STAT5 dimers that then bind to consensus sequences and promote transcription of downstream target genes, such as TNFRSF13b, MKP-1, Bcl-XL, C3ar1, Cis, Spi2.1 and Socs-1 [12, 13]. Besides proliferation- and apoptosis-related genes, STAT5 has also recently been implicated in the regulation of the expression of mammalian microRNA host genes. For example, STAT5 binds the promoter of the miR-22 host gene, and inhibition of JAK3, STAT3, and STAT5 increases expression of pri-miR-22 and miR-22, a novel tumor suppressor miRNA [14]. STAT5A/B enhances the cytokine-induced miR-193b transcription in hematopoietic stem cells (HSCs), thus providing a negative feedback for excessive signaling to restrict uncontrolled HSC expansion [15]. Despite these advances, it is largely unknown about how STAT5-regulated microRNA affects CML development.

In the present study, we detected the expression of USP15, Caspase-6, STAT5A-regulated miR-202-5p and STAT5A in CML cell lines and PBMCs of CML patients and investigated the functional relationship between these gene expression and CML cell apoptosis in the context of Imatinib or pimozide treatment. Our findings provide the first evidence that de-regulated STAT5A/miR-202-5p/USP15/Caspase-6 regulatory pathway suppresses the apoptosis of CML cells.

## Methods

The detailed procedures of cell transfection, cell viability assay, analyses of apoptosis, xenograft animal model, RNA extraction and quantitative real-time PCR, western blot analysis, in situ hybridization, vector construction and luciferase reporter assay, immunofluorescence staining, Co-immunoprecipitation assay, Chromatin immunoprecipitation (ChIP) assay and highlight sequence of miRNA as well as key reagents are described in Additional file 2.

### Patients and specimens

30 patients with chronic phase of CML (CML-CP) who were admitted to the Department of Hematology of the Second Hospital of Hebei Medical University from May 2016 to June 2017 were selected as the research objects. 30 healthy donors were selected to serve as controls. Lymphocyte separation medium was used to isolate the peripheral blood mononuclear cells (PBMCs) according to the manufacturer’s instructions. Diagnosis of Ph-positive CML was confirmed by bone marrow morphology, cytogenetic and molecular biology. Before the specimens were collected, patients did not undergo any treatment. 6 PBMCs of patients with CML blast crisis (CML-BC) (4 patients with CML-AML and 2 patients with CML-ALL) was collected from the Department of Hematology of the Second Hospital of Hebei Medical University from May 2015 to December 2018. All the patients with CML-BC were absence with Imatinib until CML progresses and didn’t have the BCR-ABL1 gene mutant. The characteristics of CML patients and healthy donors are summarized in Additional file 1: Table S2. The study protocol was approved by the Ethics Committee of Second Hospital of Hebei Medical University and written informed consent was obtained all the patients.

### Cell culture

Human CML cell lines (KCL22 and K562) were maintained in the laboratory. The Imatinib-resistant K562 cells (K562G cell line) were established as previously described [16]. KCL22 cells were cultured in Iscove’s modified Dulbecco’s medium (IMDM; Gibco, Beijing, China), with 10% fetal bovine serum (FBS) (Clark Bio, Claymont, DE, USA), 100 units/ml penicillin and 100 μg/ml streptomycin. K562, K562G cells were cultured in RPMI 1640 medium (Gibco, Beijing, China) with 10% FBS and the two antibiotics listed above. Cell lines were grown at 37 °C with 5% CO_2_. The cell lines were characterized by Genetic Testing Biotechnology Corporation (Suzhou, China) using short tandem repeat (STR) markers. Short tandem repeat analysis was used to tested *Mycoplasma* contamination.

### Target prediction and bioinformatics analysis

TargetScan (http://www.targetscan.org/vert_72/) were performed to identify the potential microRNAs target to 3’UTR of USP15. PROMO (http://alggen.lsi.upc.es) was used to search the potential transcriptional factor of pre-miR-202 and the potential element of STAT5A on the promoter region in pre-miR-202 promotor.

### Statistical analysis

Data were presented as mean ± SEM. Student’s *t* test was used to analyze differences between two groups. Spearman’s correlation analysis was used to evaluate the correlation analysis. Values of *P <* 0.05 were considered statistically significant. Graphpad Prism 7.0 software was using to perform the statistical analysis (GraphPad Software, San Diego, CA, USA).

## Results

### USP15 expression i**s significantly** down**regulated** in CML

**Fig. 1:**
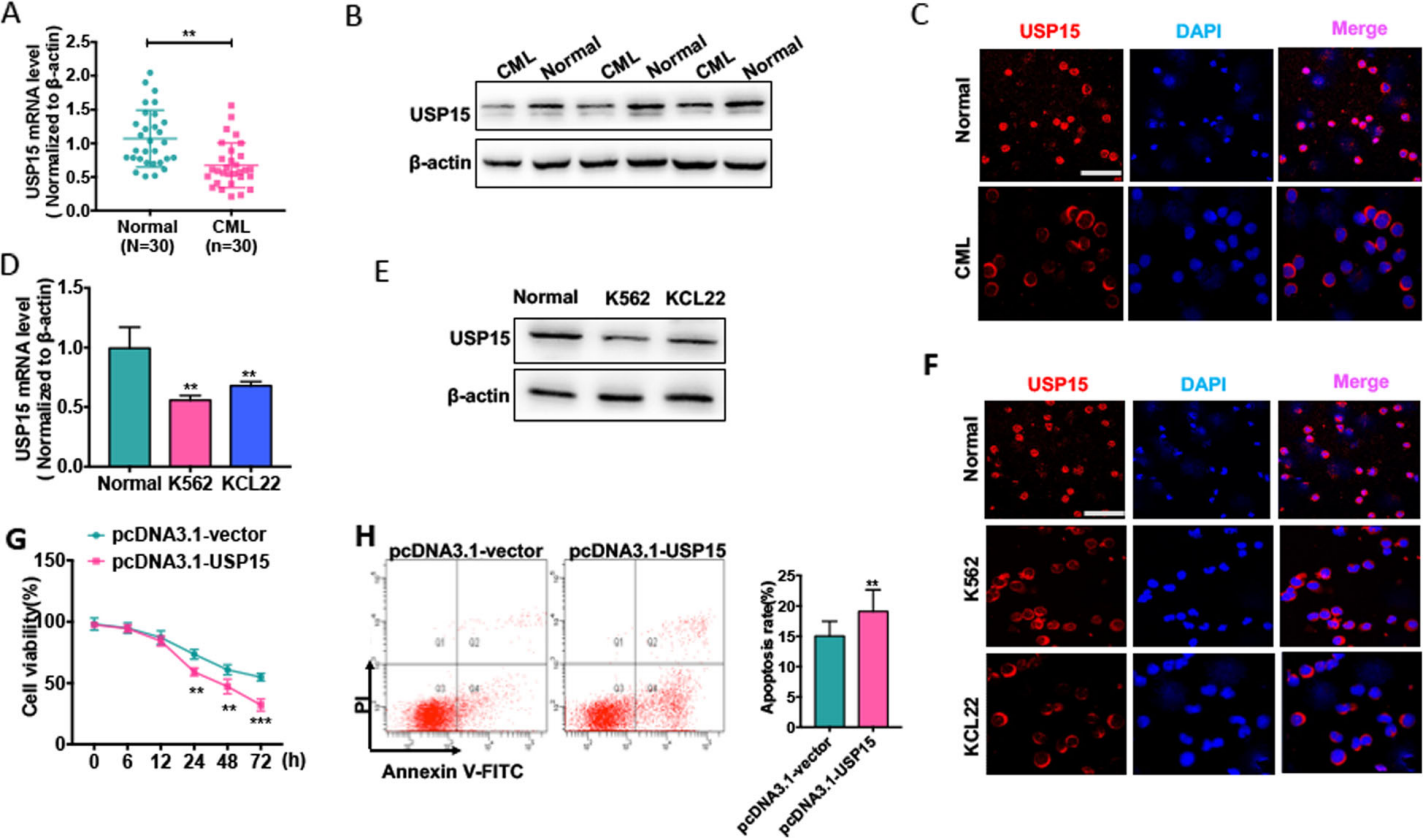
USP15 expression is significantly downregulated in CML. (**a**) qRT-PCR detected USP15 mRNA level in PBMCs of CML-CP patients (*n* = 30) and PBMCs of healthy donors (n = 30). Data are showed as mean ± ST from three independent experiments. Normalized to β-actin. **P < 0.01 vs. normal. (**b**) Western blot analysis was used to measure USP15 protein level in PBMCs of CML-CP patients (n = 30) and PBMCs of healthy donors (n = 30). The representative experiments were present. (**c**) Immunofluorescence analyzed the USP15 protein level and localization of USP15 in PBMCs of CML-CP patients and PBMCs of healthy donors. The representative results were shown. Scale bar = 64 μm. (**d**) qRT-PCR detected USP15 mRNA level in CML cell lines (K562 and KCL22) and PBMCs of healthy donors. ** *P* < 0.01 vs. normal. (**e**) Western blot analysis was used to measure USP15 protein level in CML cell lines (K562 and KCL22) and PBMCs of healthy donors. (**f**) Immunofluorescence stains analyzed the USP15 protein level and localization in CML cell lines (K562 and KCL22) compared with PBMCs of healthy donors. Scale bar = 64 μm. (**g**) K562 cells were transfected with USP15 overexpression vector pcDNA3.1-USP15 or empty vector and then cultured in different times. CCK-8 assay was used to test the cell viability. **P < 0.01, ***P < 0.001 vs pcDNA3.1-vector. (**h**) K562 cells were transfected with pcDNA4.1-USP15 or empty vector for 48 h. Cell apoptosis was detected by Annexin V-FITC/PI staining. Right panel shows the apoptosis rate from three independent experiments. **P < 0.01 vs. pcDNA3.1-vector

USP15 is previously reported to be dysregulated in many human cancers and plays critical roles in tumor development and progression [17]. Here, we first analyzed USP15 gene expression in different types of human leukemia using The Cancer Genome Atlas (TCGA) database. The results showed that the expression of USP15 was dramatically downregulated in acute leukemia including Acute Myeloid Leukemia (AML) and Acute Lymphoblastic Leukemia (ALL)comparing to the matched normal cells. A decreased USP15 expression was also found in CML but there was no significant difference between healthy donors and CML patients (Additional file 1: Fig. S1). Next, we examined USP15 mRNA and protein expression levels in PBMCs of CML-CP patients and CML cell lines. We found that USP15 mRNA level was lower in PBMCs of CML patients than in healthy donors (Fig. 1 a). Importantly, the protein level of USP15 was significantly downregulated in PBMCs of CML patients compared with healthy donors (Fig. 1 b). Immunofluorescence staining revealed that USP15 is mainly localized in the nuclei of PBMCs in healthy donors, but it existed in the cytoplasm of PBMCs and its expression level was obviously reduced in PBMCs of CML patients (Fig. 1 c). Similarly, USP15 mRNA and protein levels were downregulated in CML cell lines (K562 and KCL22), as shown by Western blotting and qRT-PCR (Fig. 1 d and e). Immunofluorescence staining also confirmed that the changes of localization and expression of USP15 in CML cell lines were very similar to those seen in PBMCs of CML patients and healthy donors, consistent with those reported previously (Fig. 1 f) [18].

To further identify whether the downregulation of USP15 is correlated with CML cell proliferation and apoptosis, we overexpressed USP15 in K562 cells (Additional file 1: Fig. S2) and examined the effects of enforced USP15 expression on K562 cells. Expectedly, overexpression of USP15 significantly decreased the cell viability and thus increased the apoptosis of K562 cells (Fig. 1 g and h). Together, these findings suggest that the downregulation of USP15 is responsible for increased CML cell proliferation and decreased apoptosis.

### Downregulation of USP15 contributes to Imatinib resistance of CML cells

**Fig. 2:**
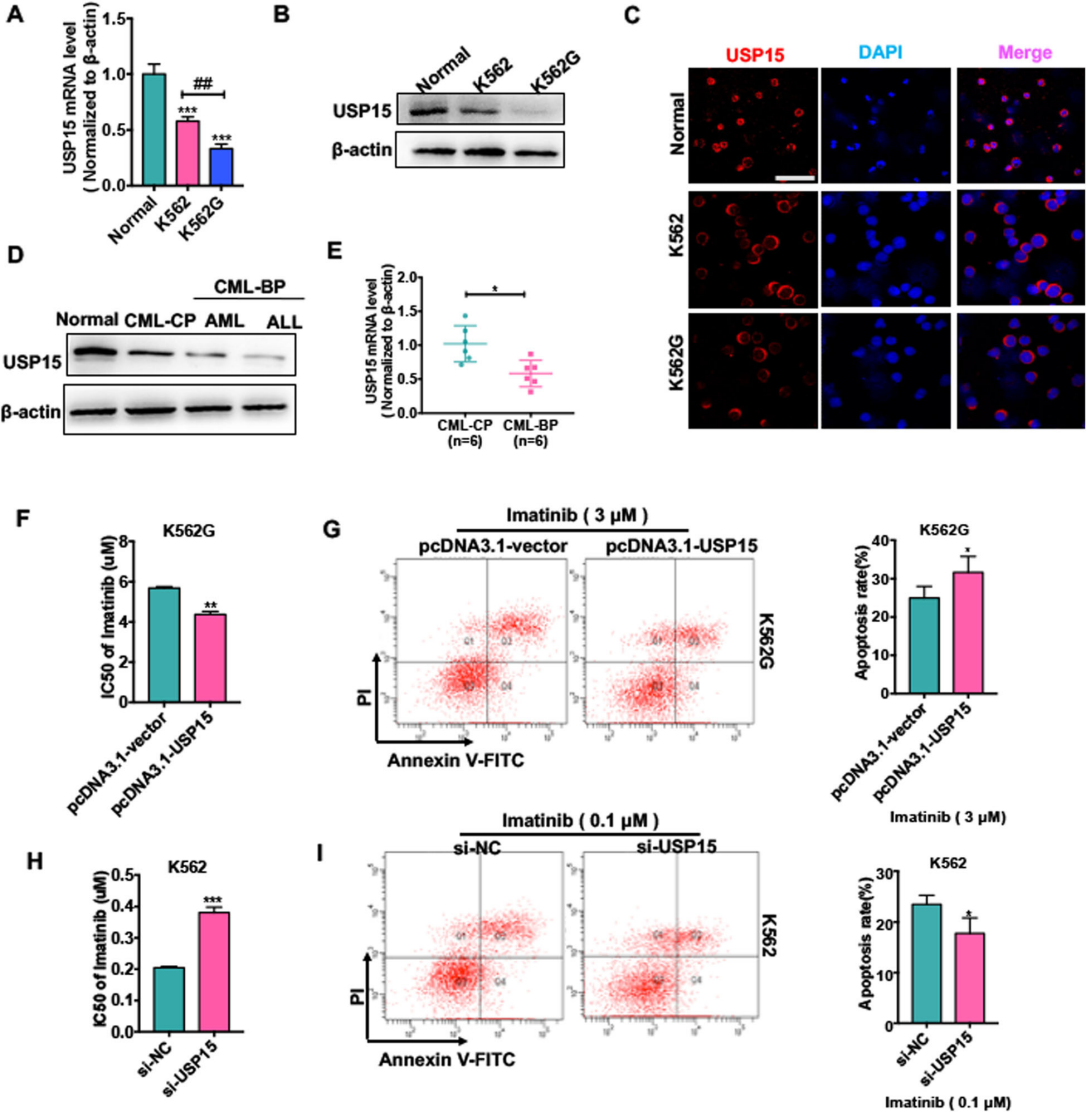
Downregulation of USP15 contributes to Imatinib resistance of CML cells. (**a**) qRT-PCR was used to detect USP15 mRNA level in K562 cells, K562G cells and PBMCs of healthy donors. *** *P* < 0.001 vs normal; ## *P* < 0.01 vs. K562 cell. (**b**) Western blot analysis was used to evaluate USP15 protein level in K562 cells, K562G cells and PBMCs of healthy donor. (**c**) Immunofluorescence analyzed the USP15 protein level and the localization of USP15 in K562 cells, K562G cells and PBMCs of healthy donor. Scale bar = 64 μm. (**d**) Western blot analysis was used to evaluate USP15 protein level in different phases of CML (CML-CP, CML-BP-AML, CML-BP-ALL) patients’ PBMCs compared with the PBMCs of normal donors. (**e**) qRT-PCR was used to test USP15 mRNA level in PBMCs of CML-CP (*n* = 6) patients and PBMCs of CML-BP patients (n = 6). *P < 0.05 vs. CML-CP. (**f**) K562G cells were transfected with pcDNA3.1-USP15 or empty and then treated with Imatinib in different concentrations for 48 h. Imatinib IC50 was evaluated by CCK-8 assay. **P < 0.01 vs. pcDNA3.1-vector. (**g**) K562G cells were transfected with pcDNA3.1-USP15 or empty vector and then treated with Imatinib (3 μm) for 48 h. Cell apoptosis was evaluated by Annexin V-FITC/PI staining. Right panel shows the analysis of apoptosis rate. *P < 0.05 vs. pcDNA3.1-vector. (**h**) K562 cells were transfected with si-USP15 or si-NC and then treated Imatinib in different concentrations for 48 h. Imatinib IC50 was evaluated by CCK-8 assay. ***P < 0.001 vs. si-NC. (**i**) K562 cells were transfected with si-USP15 or si-NC and then treated with Imatinib (0.1 μm) for 48 h. Cell apoptosis were evaluated by Annexin V-FITC/PI staining. *P < 0.05 vs si-NC

Because previously studies have reported that dysregulation of USP15 could result in paclitaxel resistance in HeLa cells [9], we wanted to investigate whether USP15 downregulation is associated with CML Imatinib resistance. As previously described, we first established an Imatinib-resistant K562 cell line (K562G cell line) and confirmed that the IC50 values of Imatinib in K562G cells were 20 folds higher than those in parental cell line (Additional file 1: Fig. S3). We next detected USP15 protein and mRNA levels in K562 and K562G cells. As shown in Fig. 2 a and b, the expression levels of USP15 mRNA and protein were much lower in K562G cells than in K562 cells. A similar result was obtained by immunofluorescence staining of USP15 (Fig. 2 c).

In addition, we analyzed the expression changes of USP15 in CML-BC patients who were confirmed to be secondary Imatinib resistance. The results showed that USP15 mRNA and protein levels were markedly decreased in PBMCs of CML-BC patients (*n* = 6) compared with age-matched CML-CP patients (n = 6) (Fig. 2 d and e). These results indicate that Imatinib-resistant cell line and CML-BC patients have a decreased level of USP15 expression. Further, loss- and gain-of-function experiments were performed to investigate whether USP15 plays a role in Imatinib resistance of CML cells. As shown in Fig. 2 f, enforced USP15 expression in K562G cells significantly decreased the IC50 values of Imatinib and facilitated Imatinib-induced cell apoptosis, as evidenced by flow cytometry (Fig. 2 g). Conversely, USP15 downregulation in K562 cells dramatically increased the IC50 values of Imatinib and attenuated Imatinib-induced cell apoptosis (Fig. 2 h and i). These results indicate that depletion of USP15 increases, while overexpression of USP15 reduces the resistance of CML cells to Imatinib.

### Downregulation of USP15 decreases the apoptosis of CML cells by lowering the level of Caspase-6 protein

**Fig. 3:**
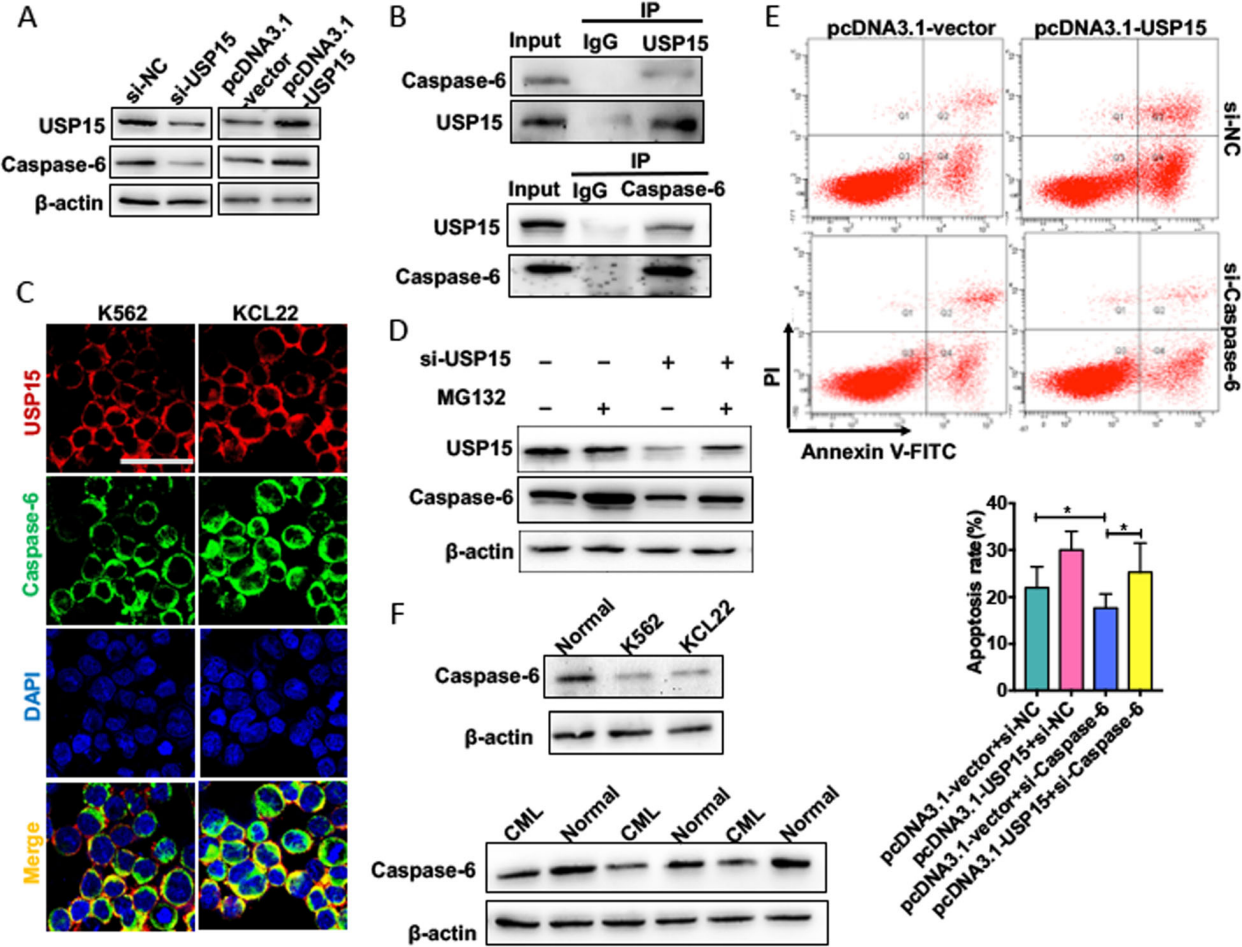
Downregulation of USP15 decreases the apoptosis of CML cells by lowering the level of Caspase-6 protein. (**a**) K562 cells were transfected with si-USP15,si-NC, pcDNA3.1-USP15 and pcDNA3.1-vector, respectively. Western blot analysis was performed to test protein levels of USP15 and Caspase-6. (**b**) Co-immunoprecipitation (CoIP) assays were performed to detect the interaction between USP15 and Caspase-6. (**c**) K562 cells were stained with anti-USP15 antibody (red) and anti-Caspase-6 antibody (green). Immunofluorescence stains analyzed the co-localization of USP15 protein and Caspase-6 protein in K562 cells. Scale bar = 64 μm. (**d**) K562 cells were transfected with si-NC or si-USP15 for 24 h then treated with MG132 for 6 h or not. USP15 and Caspase-6 protein level was detected by Western blot analysis. (**e**) K562 cells were transfected with pcDNA3.1-USP15 or si-Caspase-6, respectively, or co-transfected with pcDNA3.1-USP15 and si-Caspase-6 together. Cell apoptosis were evaluated by Annexin V-FITC/PI staining. Below panel shows the analysis of apoptosis rate. *P < 0.05 vs. corresponding control

USP15 is known to regulate the expression of multiple proteins at the post-translational level via ubiquitin-proteasome pathway [19]. To clarify the downstream proteins of USP15 in CML cells, we performed co-immunoprecipitation coupled with mass spectrometry (CoIP-MS) and found that 25 proteins might be interacted with USP15 (Additional file 1: Table S1). Among these proteins, Caspase-6, as a major activator of Caspase-8 in vivo, has a critical role in promotion of apoptosis [20] and thus aroused our attention. we first analyzed the influence of USP15 on Caspase-6 protein expression. As expected, USP15 overexpression markedly increased, whereas its knockdown reduced Caspase-6 protein level in K562 cells (Fig. 3 a). Furthermore, a co-immunoprecipitation assay showed that there existed interaction between endogenous Caspase-6 and USP15 (Fig. 3 b). Immunofluorescence staining also showed that endogenous USP15 and Caspase-6 were co-localized in the cytoplasm of K562 cells (Fig. 3 c).

To further determine whether USP15 is involved in the regulation of Caspase-6 degradation, we used proteasome inhibitor MG132 and lysosome inhibitor chloroquine (CQ) to treat K562 cells. The results showed that expression level of Caspase-6 protein was obviously increased in K562 cells treated with MG132, but not CQ, knockdown of USP15 in K562 cells partly decreased the upregulation of Caspase-6 protein level by MG132 even in the presence to MG132, indicating that the ubiquitin-proteasome pathway mediates the degradation of Caspase-6 (Fig. 3 d and Additional file 1: Fig. S4A). Additionally, K562 cells were transfected with si-USP15 or si-NC and then treated with MG132 to suppress ubiquitin-mediated degradation. Co-immunoprecipitation experiments results showed that knockdown of USP15 in K562 cells could significantly increase the of accumulation ubiquitinated Caspase-6 level compared with si-NC (Additional file 1: Fig. S4B), suggesting that USP15 positively regulates Caspase-6 protein level of by promoting de-ubiquitination of Caspase-6 and inhibiting the degradation of Caspase-6. Thereafter, flow cytometry revealed that knockdown of Caspase-6 decreased K562 cell apoptosis, and this effect could be reversed by overexpressing USP15 (Fig. 3 e). In addition, the expression level of Caspase-6 protein was significantly decreased in CML cell line and CML patients (Fig. 3 f). These findings indicate that expression level of Caspase-6, as a downstream protein of USP15, is regulated at the post-translational level by deubiquitinating activity of USP15 and that downregulation of USP15 leads to decreased Caspase-6 level.

### USP15 is a direct target of miR-202-5p

**Fig. 4:**
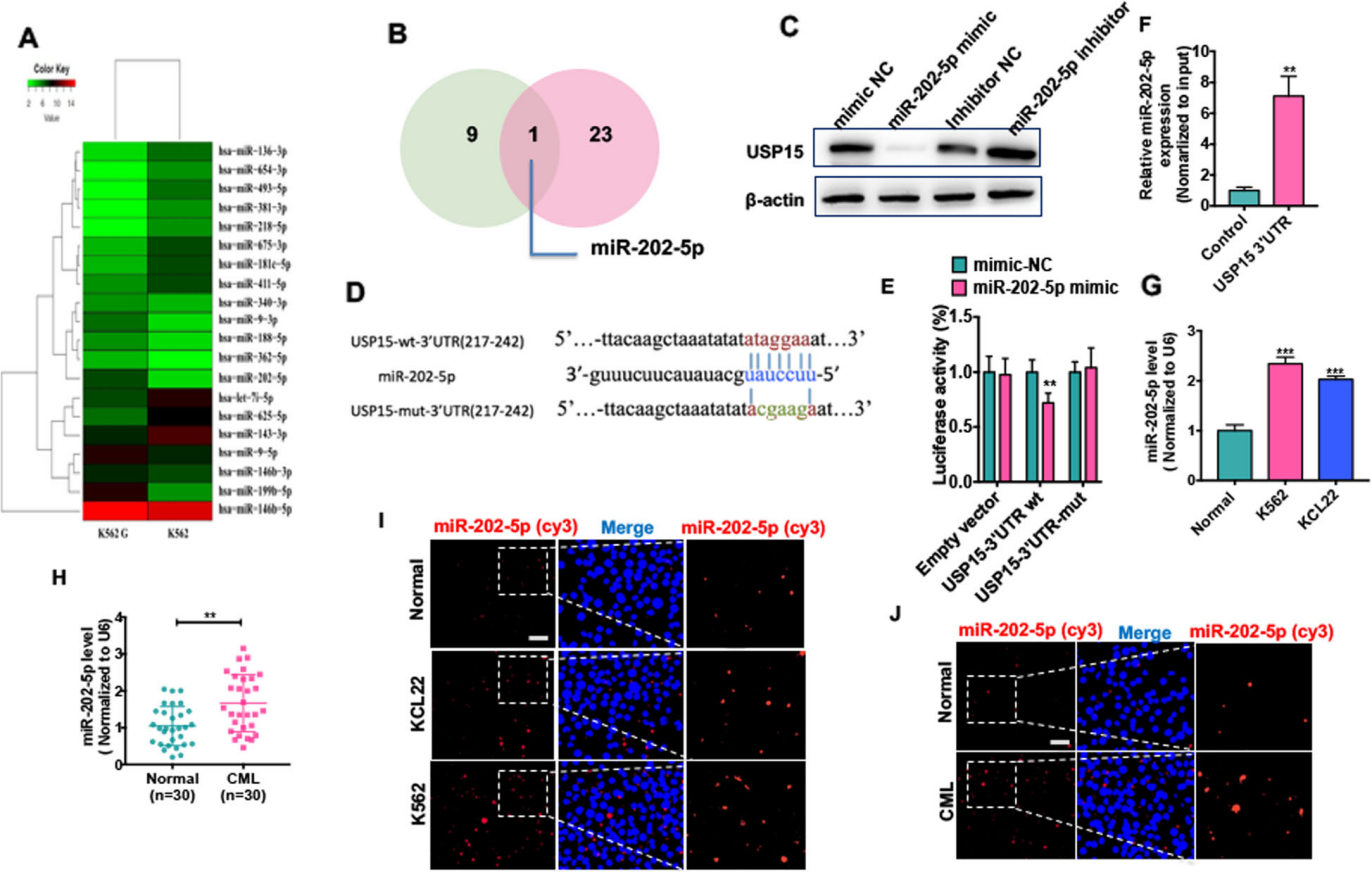
USP15 as a direct target of miR-202-5p. (**a**) Heat map showing the differential expression (fold changes) of miRNA between K562 and K562G cells from RNA-seq analysis. (**b**) Venn diagram performed that miRNA upregulation in K562G (green panel) cells overlapped the predicted miRNA ((http://www.targetscan.org/vert_72/) (red panel) which may target USP15. (**c**) K562 cells were transfected with miR-202-5p mimic, mimic-NC, miR-202-5p inhibitor or inhibitor-NC, respectively. Protein level of USP15 was measured by Western blot analysis. (**d**) Potential binding site of miR-202-5p at the 3′ UTR of USP15. (**e**) K562 cells co-transfected cells with miR-202-5p mimic and wild-type (WT) or mutant (mut) USP15 3′-UTR-luciferase reporter. Luciferase reporter assays were used to detect luciferase activity. **P < 0.01 vs. mimic-NC. (**f**) USP15 3′-UTR and control RNA with biotin-labeled uridine triphosphate were transfected into K562 cells for 24 h. The miRNAs were extracted after a pull-down assay, and miR-202-5p expression was detected by qRT-PCR. Multiple miRNAs could be pulled down by RBM5 3′-UTR. **P < 0.01 vs. control RNA. (**g**) qRT-PCR was used to test miR-202-5p level in CML cell lines (K562 and KCL22) and PBMCs of healthy donors. Normalized to U6. ***P < 0.001 vs. normal (**h**) qRT-PCR detected the expression of miR-202-5p expression in PBMCs of CML-CP (*n* = 30) patients compared with PBMCs of healthy honors (*n* = 30). **P < 0.01 vs. normal. (**i**) Fluorescence in situ hybridization (FISH) detected the miR-202-5p in CML cell lines and PBMCs of healthy donors. Blue staining represents the nucleus and red staining indicates miR-202-5p. Scale bar = 20 μm. (**j**) FISH detected the miR-202-5p in PBMCs of CML-CP patients and PBMCs of healthy donors. Scale bar = 20 μm

**Fig. 5:**
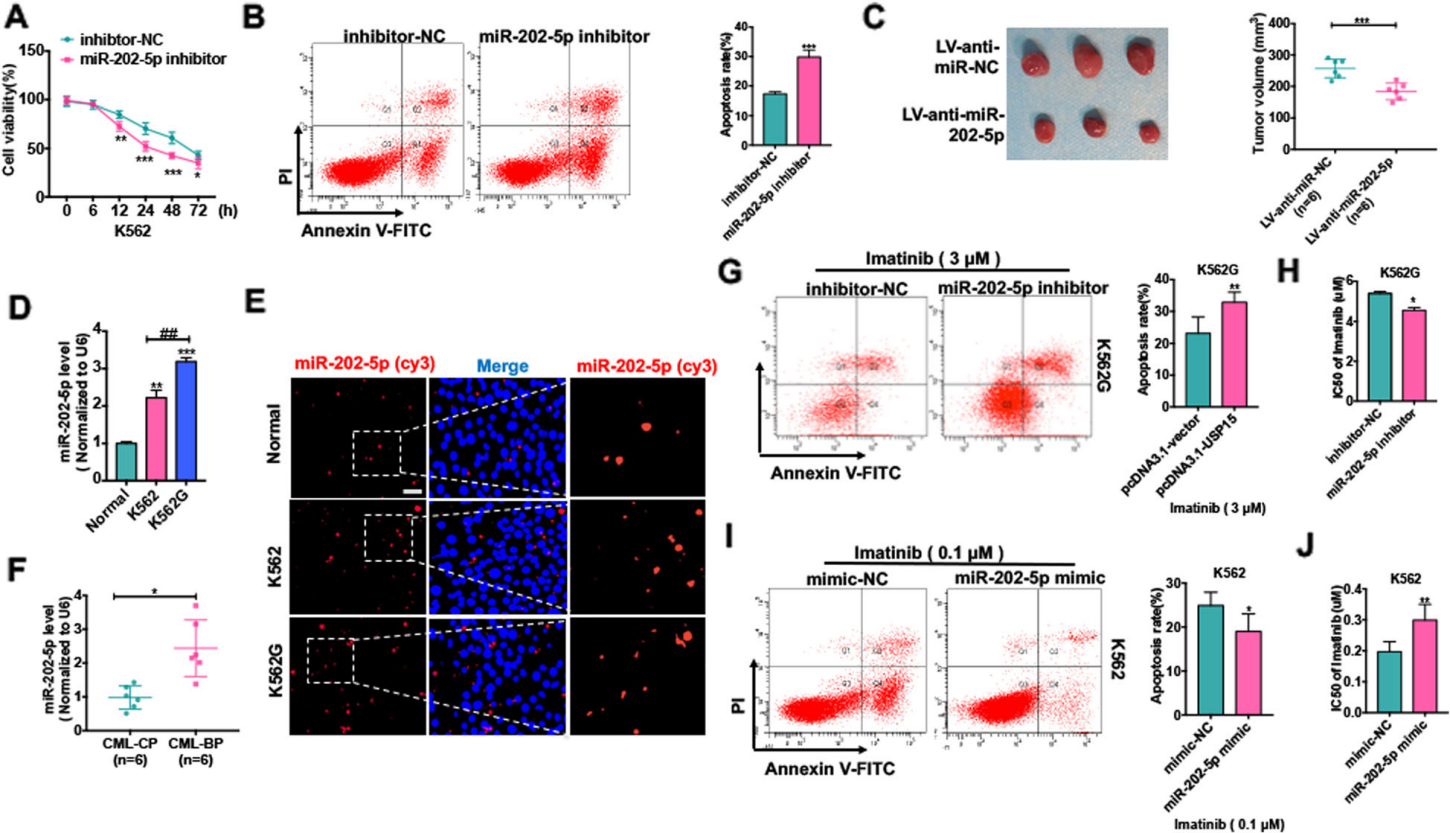
Upregulation of miR-202-5p enhances the resistance of CML cells to Imatinib by inhibiting the apoptosis of CML cells. (**a**) K562 cells were transfected with miR-202-5p inhibitor or inhibitor-NC Cells and cultured in different times. CCK-8 assay was used to test the cell viability. *P < 0.05, **P < 0.01, **P < 0.001 vs. inhibitor-NC. (**b**) K562 cells were transfected with miR-202-5p inhibitor or inhibitor-NC for 48 h. Cell apoptosis were test by Annexin V-FITC/PI staining. **P < 0.01 vs. inhibitor-NC. (**c**) Stably express anti-miR-202-5p K562 cells or negative control K562 cells were injected subcutaneously in 200 μl 1640/Matrigel (100: 100) into the right posterior ankle of the nude mice to establish xenograft tumors (each group, *n* = 6). Tumor volumes were monitored by direct measurement with calipers and calculated by the formula: (length × width^2^)/2. ***P < 0.001 vs. LV-miR-NC. (**d**) qRT-PCR was used to detect miR-202-5p level in K562 cells, K562G cells and PBMCs of healthy donors. ** *P* < 0.01, *** *P* < 0.001 vs normal, ## *P* < 0.01 vs. K562 cell. (**e**) FISH assay was used to analyzed miR-202-5p level and localization in K562 cells and K562G cells compared with PBMCs of healthy donors. Scale bar = 20 μm. (**f**) qRT-PCR was used to detect miR-202-5p level in PBMCs of CML-CP (n = 6) patients and PBMCs of CML-BC patients (n = 6) *P < 0.05 vs. CML-CP. (**g**) K562G cells were transfected with miR-202-5p inhibitor or inhibitor-NC and then treated with Imatinib (3 μM) for 48 h. Cell apoptosis were evaluated by Annexin V-FITC/PI staining. **P < 0.01 vs. inhibitor NC. (**h**) K562G cells were transfected with miR-202-5p inhibitor or inhibitor-NC and then treated with Imatinib in different concentrations for 48 h. Imatinib IC50 of K562G cells was evaluated by CCK-8 assay. *P < 0.05 vs inhibitor-NC. (**i**) K562 cells were transfected with miR-202-5p mimic or mimic and then treated with Imatinib (0.1 μM) for 48 h. Cell apoptosis were evaluated by Annexin V-FITC/PI staining. *P < 0.01 vs. mimic-NC. (**j**) K562 cells were treated with Imatinib in different concentrations for 48 h. Imatinib IC50 of K562 cells was evaluated by CCK-8 assay. **P < 0.01 vs mimic-NC

Because we found that USP15 downregulation led to decreased apoptosis in CML cells by lowering the level of Caspase-6 protein, we sought to know whether the expression of USP15 is suppressed in CML cells by a microRNA that targets 3′-untranslated region (3′-UTR) of USP15 mRNA. To do this, high-throughput sequencing analysis of microRNAs was performed in K562G cells versus its parental cells. The results showed that 9 and 11 miRNAs were significantly upregulated and downregulated, respectively, in K562G cells (Fig. 4 a). miRNA target predictions by TargetScan (http://www.targetscan.org/vert_72/) were used to screen putative miRNAs that may target USP15 3′-UTR. Notably, miR-202-5p was upregulated in K562G cells, and 3′-UTR of USP15 mRNA contains highly conserved miR-202-5p-binding site (Fig. 4 b). To determine whether miR-202-5p regulates USP15 expression in CML cells, we transfected K562 cells with miR-202-5p mimic/inhibitor to overexpress/knock down miR-202-5p. qRT-PCR results showed that miR-202-5p mimic significantly increased, while its inhibitor obviously reduced miR-202-5p expression level in K562 cells (Additional file 1: Fig. S5). Using loss- and gain-of-function experiments, we found that miR-202-5p mimic dramatically decreased, and miR-202-5p inhibitor markedly increased USP15 protein level (Fig. 4 c). In further experiments, we used luciferase reporter assay to detect the effect of miR-202-5p on USP15 expression. As indicated in Fig. 4 d, there existed a putative binding site of miR-202-5p in 3′-UTR of USP15 mRNA. Transfecting K562 cells with miR-202-5p mimic, but not mimic-NC, obviously decreased luciferase activity driven by the wild type of USP15 3′-UTR. However, luciferase activity directed by the mutant-type of USP15 3′-UTR was not affected by miR-202-5p mimic (Fig. 4 e). Then we used a T7 RNA transcriptase to generate USP15 3′-UTR in vitro with biotin-labeled uracil. The USP15 3′-UTR was transfected into K562 cells, and miR-202-5p was enriched via the pull-down method. As shown in Fig. 5 f, miR-202-5p was significantly enriched in the precipitates by qRT-PCR, indicating that miR-202-5p could bind to USP15 3’UTR and regulate USP15 expression.

To investigate the clinical significance of miR-202-5p, we analyzed the expression level of miR-202-5p in CML patients and CML cell lines. The results showed that miR-202-5p expression was much higher in K562 and KCL22 cell lines than in PBMCs of healthy donors (Fig. 4 g). This was also confirmed in PBMCs of CML-CP patients by qRT-PCR (Fig. 4 h). Fluorescent in situ hybridization (FISH) analysis showed that miR-202-5p obviously increased in CML cell lines and PBMCs of CML patients and was localized in the cytoplasm of these cells (Fig. 4 i and j).

### Upregulation of miR-202-5p enhances the resistance of CML cells to Imatinib by inhibiting the apoptosis of CML cells

Considering that miR-202-5p is increased in CML cells, its impact on CML cell proliferation and apoptosis was evaluated. We knocked down miR-202-5p in K562 cells by miR-202-5p inhibitor and found that depletion of miR-202-5p dramatically inhibited cell proliferation and increased apoptosis compared with those transfected with inhibitor-NC (Fig. 5 a and b). Further, the K562 cells stably expressing anti-miR-202-5p were implanted into nude mice to observe the effect of miR-202-5p depletion on K562 cell xenograft growth. As a result, tumor volume was significantly reduced in miR-202-5p-depleted mice by LV-anti-miR-202-5p compared with control mice (Fig. 5 c). These findings indicate that upregulation of miR-202-5p results in an increased proliferation and decreased apoptosis in CML cells.

To further clarify whether Imatinib resistance of CML cells is related to miR-202-5p upregulation, we detected the expression level of miR-202-5p in K562G and K562 cells. qRT-PCR and FISH analysis showed that miR-202-5p expression level was much higher in K562G cells than in K562 cells (Fig. 5 d and e). Similarly, miR-202-5p level was also significantly upregulated in CML-BC patients compared with that in CML-CP patients (Fig. 5 f). Next, we depleted miR-202-5p in K562G cells by miR-202-5p inhibitor and found that depletion of miR-202-5p decreased the IC50 values of Imatinib, thus facilitating Imatinib-induced apoptosis of K562G cells (Fig. 5 g and h). In contrast, transfection of K562 cells with miR-202-5p mimic increased the IC50 values of Imatinib and inhibited K562 cell apoptosis induced by Imatinib (Fig. 5 i and j). These findings indicate that upregulation of miR-202-5p enhances the resistance of CML cells to Imatinib by inhibiting cell apoptosis.

### STAT5A is upregulated in CML cells and directly activates miR-202-5p transcription by binding to **the** pre-miR-202 promoter

**Fig. 6:**
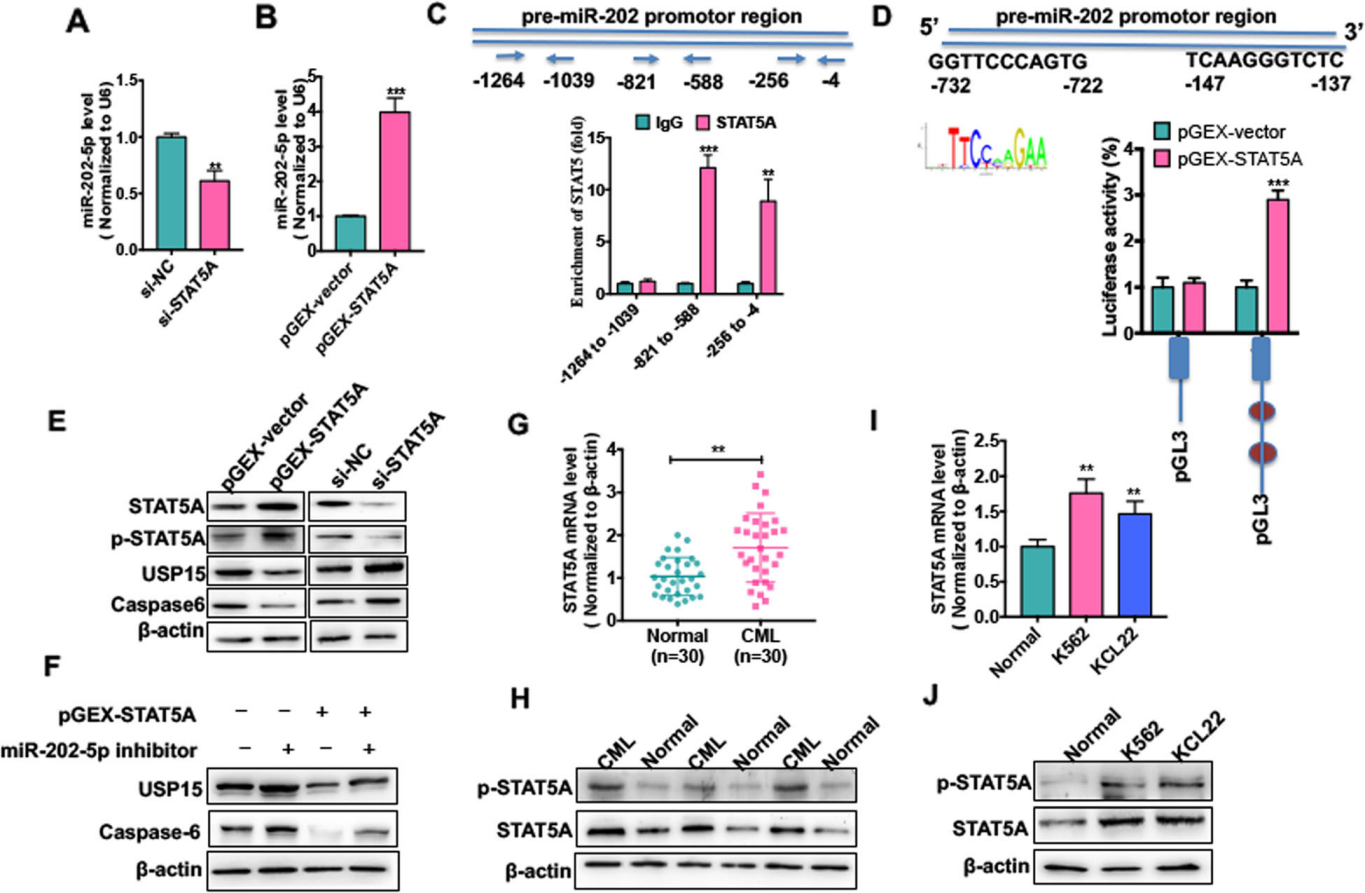
STAT5A is upregulated in CML cells and directly activates miR-202-5p transcription by binding to the pre-miR-202 promoter. (**a**) K562 cells were transfected with si-STAT5A or si-NC. miR-202-5p level was detected by qRT-PCR. ** *P* < 0.01 vs si-NC. (**b**) K562 cells were transfected with pGEX-STAT5 or pGEX-vector. miR-202-5p level was detected by qRT-PCR. *** *P* < 0.001 vs pGEX-vector. (**c**) ChIP-qPCR was used to detect STAT5A binding to the pre-miR-202 promoter regions in K562 cells. Three primers of indicate positions was used for ChIP-PCR analysis.**P < 0.01, ***P < 0.001 vs. IgG. (**d**) Pre-miR-202 promoter-luciferase reporters were co-transfected with pGEX-STAT5A expression vector into K562 cells, and luciferase reporter assays were performed. *** *P* < 0.001 vs. pGEX-vector. (**e**) K562 cells were transfected with pGEX-STAT5A, pGEX-vector, si-STAT5A and si-NC, respectively. Protein levels of STAT5A, pSTAT5A, USP15 and Caspase-6 were detected by Western blot analysis. (**f**) K562 cells were transfected with pGEX-STAT5A or miR-202-5p inhibitor respectively, or co-transfected with them together. USP15 and caspase6 protein level was detected by Western blot analysis. (**g**) qRT-PCR detected STAT5A mRNA level in PBMCs of CML-CP patients (n = 30) and PBMCs of healthy donors (n = 30). **P < 0.01 vs. normal. (**h**) Western blot analysis was used to measure protein levels of STAT5A and p-STAT5A in PBMCs of CML-CP patients (n = 30) and PBMCs of healthy donors (n = 30). The representative experiments were present. (I) qRT-PCR detected STAT5A mRNA level in CML cell lines (K562 and KCL22) and PBMCs of healthy donors. **P < 0.01 vs. normal. (**j**) Western blot analysis was used to measure protein levels of STAT5A and p-STAT5A in CML cell lines (K562 and KCL22) and PBMCs of healthy donors

To investigate the mechanism underlying upregulation of miR-202-5p in CML cells, we used an online software PROMO (http://alggen.lsi.upc.es) to predict the putative transcriptional factors that can bind to the pre-miR-202 promoter. The results revealed that STAT5A, WT1 [21] and CEBPA [22], all of which can be activated by BCR-ABL, were predicted to be capable of binding to the pre-miR-202 promoter. Next, we examined the effect of these three transcriptional factors on pre-miR-202-5p expression. As shown in Additional file 1: Fig. S6, knockdown of STAT5A by its specific si-RNA significantly decreased pre-miR-202-5p level in K562 cells, however, knockdown of CEBPA and WT1 did not affect pre-miR-202-5p expression. Thus, we focused our attention on STAT5A in the subsequent experiments. First, we demonstrated that overexpression or knockdown of STAT5A in K562 cells significantly increased or decreased both total STAT5 and p-STAT5A level (Additional file 1: Fig. S7). Then, qRT-PCR results showed that overexpression or knockdown of STAT5A also significantly upregulated or suppressed, respectively, miR-202-5p expression compared with their corresponding control (Fig. 6 a and b). To provide additional confirmation that STAT5A directly activates pre-miR-202-5p transcription, we used PROMO prediction software to identify the putative binding sites of STAT5A in the pre-miR-202 promoter and found that 3 putative binding sites existed in the pre-miR-202 promoter region. ChIP-PCR analysis showed that STAT5A was directly bound to the − 4 to − 256 bp and the − 588 to − 821 bp region of the pre-miR-202 promoter (Fig. 6 c). Furthermore, luciferase activity assay revealed that co-transfecting K562 cells with STAT5A expression vector and pre-miR-202 promoter-directed luciferase reporter significantly increased luciferase activity compared with that transfected with empty vector (Fig. 6 d).

In the further experiments, we determined whether STAT5A affects the expression of USP15 and Caspase-6. The results showed that enforced STAT5A expression in K562 cells markedly attenuated the expression level of USP15 and Caspase-6, whereas knockdown of STAT5A had the opposite effects (Fig. 6 e). Additionally, we found that depletion of endogens miR-202-5p by miR-202-5p inhibitor could abrogate the inhibitory effect of STAT5A overexpression on USP15 and Caspase-6 expression (Fig. 6 f), indicating that miR-202-5p mediates STAT5A repression of USP15 expression. Additionally, CML patients and cell lines had a lower mRNA expression level than the healthy PBMCs (Fig. 6 g and h). Protein level of STAT5A and p-STAT5A was also increased in CML patients and cell lines detected by Western blotting (Fig. 6 i and j). These findings suggest that STAT5A positively regulates miR-202-5p expression by binding to the promoter of the miR-202-5p precursor.

### Pimozide induces CML cell apoptosis by blocking STAT5A/miR-202-5p/USP15/Caspase-6 regulatory pathway

**Fig. 7:**
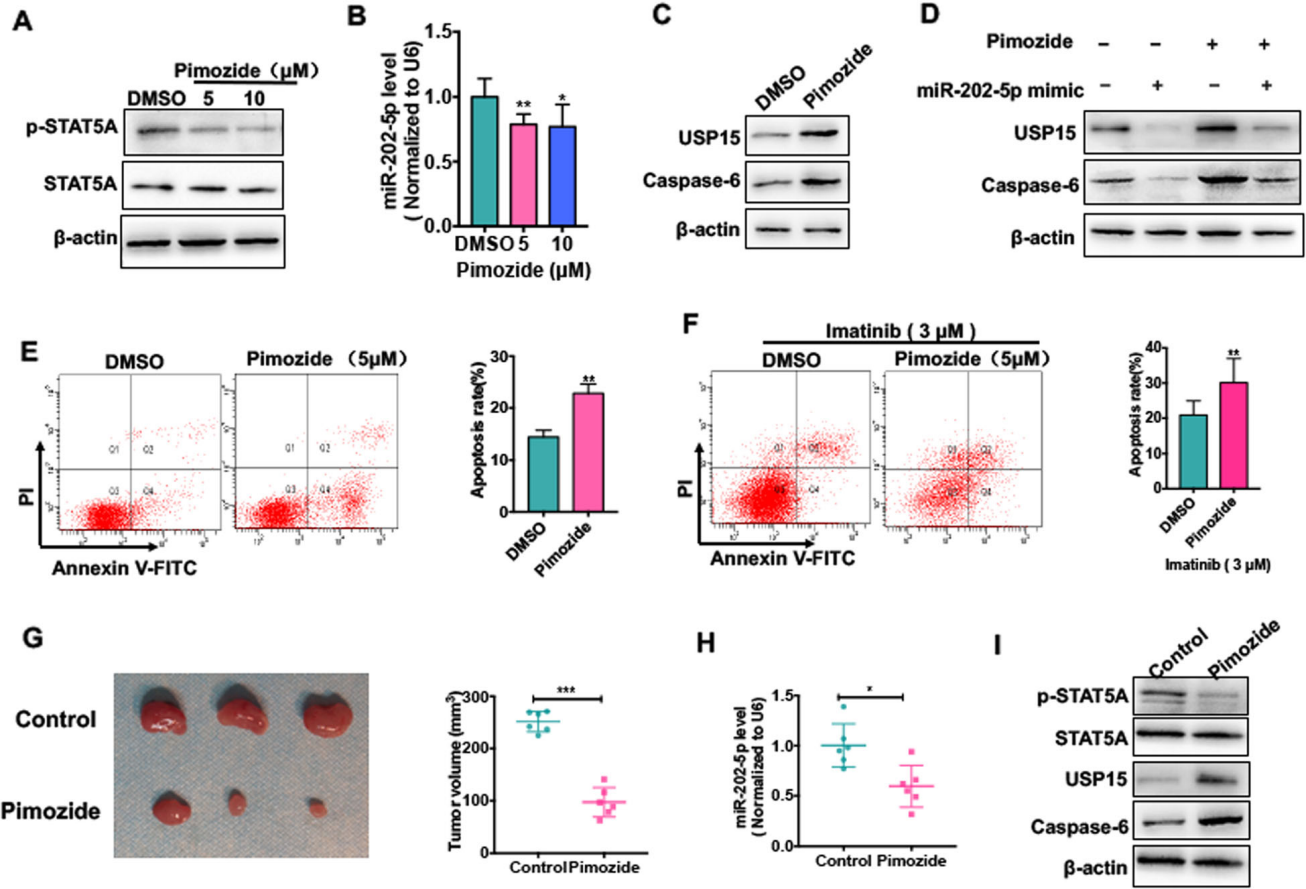
Pimozide induces CML cell apoptosis by blocking STAT5A/miR-202-5p/USP15/Caspase-6 regulatory pathway. (**a**) K562 cells were treated with Pimozide (5 or 10 μM) for 48 h. Western blot analysis was used to detect protein levels of STAT5A and p-STAT5A. (**b**) K562 cells were prepared as (**a**), qRT-PCR was used to test miR-202-5p expression level. * *P* < 0.05, ** *P* < 0.01 vs DMSO control. (**c**) K562 cells were treated with Pimozide (5 μM) for 48 h. Western blot analysis was used to detect protein levels of USP15 and Caspase-6. (**d**) K562 cell were transfected with miR-202-5p mimic or mimic-NC and then treated with Pimozide (5 μM) or not. Western blot analysis was used to detect protein levels of USP15 and Caspase-6. (**e**) K562 cells were prepared as (**c**), Annexin V-FITC/PI staining was used to detect cell apoptosis. Right panel shows the apoptosis rate. ** *P* < 0.01 vs DMSO control. (**f**) K562G cells were treated with Imatinib (3 μM) and combination of Pimozide (5 μM) or not for 48 h. Annexin V-FITC/PI staining was used to detect cell apoptosis. Right panel shows the apoptosis rate. **P < 0.01 vs. DMSO control. (**g**) K562 cells were injected into the right posterior ankle of the nude mice to establish xenograft tumors. Mice were treated with Pimozide or not. Tumor volume was measured. ***P < 0.001 vs. control. (**h**) RNA was extracted from excised tumors and the miR-202-5p level was determined by qRT-PCR. *P < 0.05 vs. control. (**i**) Total proteins were extracted from excised tumors and the expression protein levels of STAT5, p-STAT5, USP15 and Caspase-6 were determined by Western blot analysis

Previous study showed that pimozide, a neuroleptic drug, potently induces apoptosis in CML cells by inhibiting STAT5 activity [23]. In this study, our results indicated that STAT5A upregulation in CML cells facilitates the formation of miR-202-5p/USP15/Caspase-6 regulatory axis, thus suppressing the apoptosis of CML cells. Here, we sought to determine whether pimozide promotes CML cell apoptosis by regulating STAT5/miR-202-5p/USP15/Caspase-6 axis. First, we showed that the level of phosphorylated STAT5A (p-STAT5A) was remarkedly reduced in pimozide-treated K562 cells without affecting the total STAT5 protein level (Fig. 7 a). Further, treating K562 cells with pimozide also significantly decreased miR-202-5p expression level compared with DMSO control (Fig. 7 b). Correspondingly, pimozide enhanced the expression level of USP15 and Caspase-6 protein, as shown by Western blotting (Fig. 7 c), and miR-202-5p mimic abolished the upregulation of USP15 and Caspase-6 induced by pimozide (Fig. 7 d), indicating that pimozide can block STAT5A/miR-202-5p/USP15/Caspase-6 regulatory pathway via inhibiting the phosphorylation of STAT5A.

**Fig. 8:**
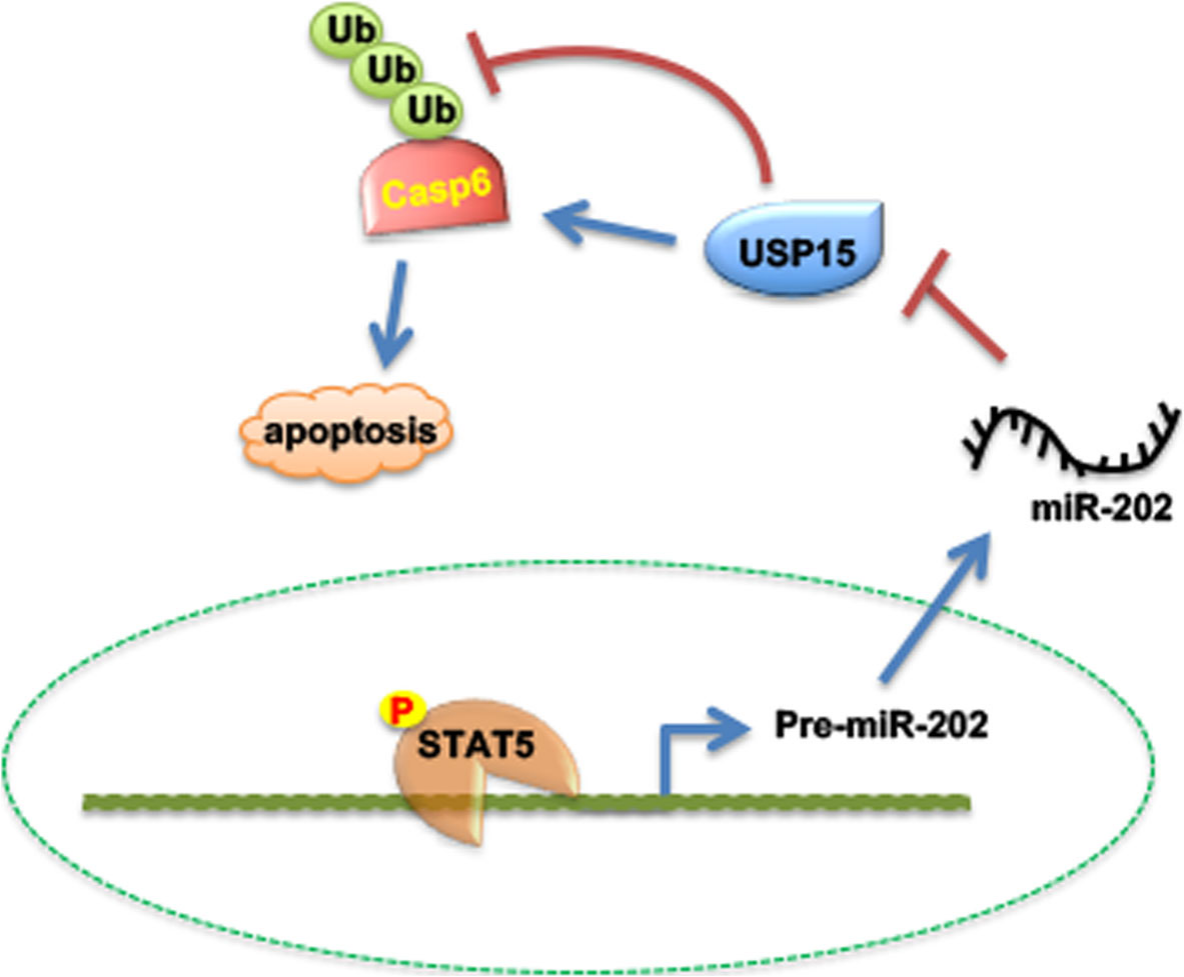
Proposal model of STAT5A/miR-202-5p/USP15/Caspase-6 regulatory axis in CML cell apoptosis

Next, the effect of pimozide on CML cell apoptosis was evaluated. As expected, pimozide significantly induced K562 cell apoptosis compared with DMSO control (Fig. 7 e). More importantly, treating K562G cells with Imatinib combined with pimozide could obviously increase the apoptosis rate of K562G cells compared with Imatinib alone (Fig. 7 f). Finally, in vivo experiments showed that pimozide dramatically reduced tumor volume in K562 cell xenograft model (Fig. 7 g). At the same time, we also detected miR-202-5p expression in the xenograft tumors. The results showed that pimozide significantly decreased the expression level of miR-202-5p compared with control group (Fig. 7 h). Similarly, in K562 cell xenograft tumors, pimozide also obviously suppressed STAT5A phosphorylation and markedly enhanced the expression of USP15 and Caspase-6, thus facilitating CML cell apoptosis (Fig. 7 i). These data again suggest that pimozide exerts its anti-leukemia effects by inhibiting STAT5A phosphorylation and thus blocking STAT5A/miR-202-5p/USP15/Caspase-6 regulatory pathway. As proposal model shown in Fig. 8, we found dysregulation of STAT5A/miR-202-5p/USP15/Caspase-6 regulatory axis contributed to CML cell apoptosis and Imatinib resistance.

## Discussion

Imatinib mesylate, the first tyrosine kinase inhibitors (TKIs) targeting BCR-ABL1, have effectively improved the survival of patients with CML [24]. However, 20–30% of CML patients haven’t been benefited from TKI treatment commonly due to TKIs resistance which lead to disease relapse and progression [25]. Most resistance of TKIs can be mediated by mutations within BCR-ABL kinase domain that interfere with ATP binding site [26]. However, approximately 50% Imatinib-resistant CML patients has not mutations in ABL domain. Therefore, BCR-ABL-independent Imatinib-resistant mechanisms may also play a role in progressive disease [27]. Previously studies have reported that the abnormal activation of pathway or gene expression may be associated with Imatinib resistance. For example, Moshe showed that LYN kinase was overexpressed and activation in Imatinib-resistant K562 cells, suggesting that expression or activation of a SRC-family kinase played a role in Imatinib resistance. Liu revealed TKI-resistant CML primary cells and cell line had a higher level of PKM2 level. Knockdown of PKM2 expression reduced cell proliferation, and induced cell apoptosis of TKI-resistant cell line after treated with Imatinib by regulation of glucose metabolism [28]. Besides, Salvatore revealed 24 proteins were overexpressed in Imatinib-resistant KCL22 compared with its parental cells and some of these proteins were involved cell-cycle pathway and anti-apoptotic pathways. In the present study, we first confirmed that USP15 is downregulated in CML cell lines and PBMCs of patients with CML-CP. The in vivo and in vitro experiments revealed overexpression of USP15 significantly reduced the cell viability and thus induced the apoptosis of K562 cells. Furthermore, Imatinib resistant K562G cells and patients with Imatinib resistance had much lower expression level of USP15. Depletion of USP15 increased, while overexpression of USP15 reduced the resistance of CML cells to Imatinib, suggesting that USP15 may function as a tumor suppressor in CML and involve in Imatinib resistance.

The ubiquitin–proteasome pathway is a critical mechanism for regulating the protein degradation [29]. Deubiquitinases (DUBs) are a group of enzymes that could remove ubiquitin chains from ubiquitinated proteins, leading to prevention the target proteins from degradation [30]. The ubiquitin-specific peptidases (USPs) are the main members of the deubiquitinase family. Increasing evidence have indicated that dysregulation of USPs contributes to multiple tumor progressions, including hematological malignancies [31]. For example, Kitamura showed the functional role of USP7 in the deubiquitination and stabilization of ASXL1 which is an independent poor prognostic factor among patients with MDS. Depletion of USP7 in HL-60 cells decreased ASXL1 protein level, suggesting that USP7 inhibitor may a potential target in AML and MDS treatment [32]. Jin reported that USP7 was overexpressed in T-ALL cells and suppression of USP7 led to significantly decrease T-ALL cell growth in vitro and in vivo by controlling the NOTCH1 protein level through deubiquitination [33]. Liu found that USP10 acted as the DUBs of SKP2 which may also promote leukemogenesis and stabilize SKP2 protein in CML cells. The in vivo and in vitro experiments confirmed that inhibition of USP10 suppressed the proliferation of CML [34]. In the present study, we found that 25 proteins might be interacted with USP15 analyzed by CoIP with mass spectrometry analysis. We further demonstrated Caspase-6, as a downstream protein of USP15, was regulated at the post-translational level by deubiquitinating activity of USP15. Caspase-6 protein level was positively regulated by USP15 and attributed to the apoptosis induced by USP15 in CML cells. However, as the deubiquitinases, USP15 may regulate more proteins degradation by deubiquitination. Further researches need to clarify the more downstream gene of USP15 and their biological functions.

These results raise the important questions about how USP15 expression is regulated in CML cells. Mechanisms controlling genes expression include transcript regulation, mutations of gene, aberrations in gene copy number, epigenetic mechanisms and micro-RNA (miRNA) regulation. miRNAs are small non-coding RNAs which negatively regulate gene expression in post-translational level through the degradation of transcripts or by interfering with protein translation [35]. It is widely accepted that abnormal expression of miRNAs may be responsible for the tumor development by targeting oncogene or tumor suppressor [36], including USPs. For example, in melanoma, overexpression of miR-593-3p led to the decrease USP13 level involved in melanoma metastasis [37]. In endocrine-resistant breast cancer, USP19 regulated by miR-15a may response to the drug resistance. In osteosarcoma, USP1 level was upregulated in osteosarcoma tissues and cell lines and due to the regulation of miR-192-5p [38]. In colorectal cancer, USP13 targeted by miR-224 and indirectly regulated smad4 gene level in colorectal cancer cells [39]. In the present study, we revealed that miR-202-5p which was overexpression in CML cell lines and patients directly targeted USP15 3’UTR and negatively regulated USP15 protein level in CML cells. Subsequently, miR-202-5p as an onco-miRNA, was responsible to CML cell proliferation and apoptosis. Importantly, miR-202-5p was higher in Imatinib-resistant CML cells and played a role in Imatinib-resistance, suggesting that miR-202-5p might be a potential biomarker in Imatinib resistance. Nevertheless, one miRNA may suppress multiple gene expression, while altered gene expression may also be affected by a variety of factors. In the present study, we found a novel mechanism for USP15 depletion in CML and Imatinib resistance was due to the overexpression of miR-202-5p. Other unknown regulations of USP15 depletion in CML requires more in vivo and in vitro experiments.

In the present study, we further demonstrated that the STAT5A crosslinked into pre-miR-202 promotor element and transcriptional up-regulated miR-202-5p expression, as evidenced by luciferase reporter gene assays and ChIP analysis. It has been clearly known that STAT5A is a master downstream effector of BCR-ABL [40]. Altered activation of STAT5A which affected by BCR-ABL tyrosine kinase can influence the CML development and progression [41]. Consistent with previously described [42], we found significantly increased expression level of STAT5 in CML cell lines and patients, especially in Imatinib-resistant patients and CML cell line. Besides, the new downstream axis of STAT5A was explored in the study. Depletion of STAT5A could dramatically increase USP15 and Caspase-6 expression levels in CML cells mediating by miR-202-5p and associated with CML apoptosis and IM resistance. That is to say, initiating from abnormal expression and activation of STAT5A, dysregulation of STAT5A/miR-202-5p/USP15/Caspase-6 regulatory axis exerts an critical role in CML cells apoptosis Imatinib resistance. Importantly, we proved that the Pimozide, as the STAT5A inhibitor, could effectively reduce the phosphorylation of STAT5A thus inhibit the activation of STAT5A/miR-202-5p/USP15/Caspase-6 regulatory axis pathway. Stimulation of Pimozide in CML cells could obviously increase USP15 and Caspase-6 expression levels, indicating that Pimozide functioned as USP15 activator though suppressing miR-202-5p level. Furthermore, our findings substantiated that Pimozide promoted CML cell apoptosis and inhibited cell growth in vivo and in vitro, consistent with the previously report [23, 43]. Subsequently, Pimozide enhanced Imatinib sensitivity in Imatinib resistant K562 cells, suggesting Pimozide may be a promising adjuvant to treat patients with CML.

## Conclusion

In conclusion, as proposal model shown in Fig. 8, we found dysregulation of STAT5A/miR-202-5p/USP15/Caspase-6 regulatory axis contributed to CML cell apoptosis and Imatinib resistance. Hence, targeting this pathway may can be a novel treatment for CML and overcoming Imatinib resistance.

## Additional files

**Additional file 1.** Supplementary Figures and Tables.

**Additional file 2.** Supplemental Experimental Procedures.

## Data Availability

Not applicable.

## Abbreviations

ALL: Acute lymphoblastic leukemia;
ChIP: Chromatin immunoprecipitation;
CML: Chronic myeloid leukemia;
CoIP: Co-immunoprecipitation;
DUBs: Deubiquitinases;
PI3K: Phosphoinositide 3-kinase;
RT-qPCR: Reverse transcription-quantitative polymerase chain reaction;
STAT5: Signal transducer and activator of transcription 5;
TCGA: The Cancer Genome Atlas;
TKIs: Tyrosine kinase inhibitors
USPs: Ubiquitin-specific peptidases;
UTR: untranslated region;
