# Supplementary material for: De-regulated STAT5A/miR-202-5p/USP15/Caspase-6 regulatory axis suppresses CML cell apoptosis and contributes to Imatinib resistance
Source: J Exp Clin Cancer Res. 2020 Jan 17;39:17. doi: 10.1186/s13046-019-1502-7 (PMC6969434; doi:10.1186/s13046-019-1502-7)
Supplement: Supplementary file 1 — Additional file 1. Supplementary Figures and Tables. [file 13046_2019_1502_MOESM1_ESM.pdf]

Supplementary Material

A

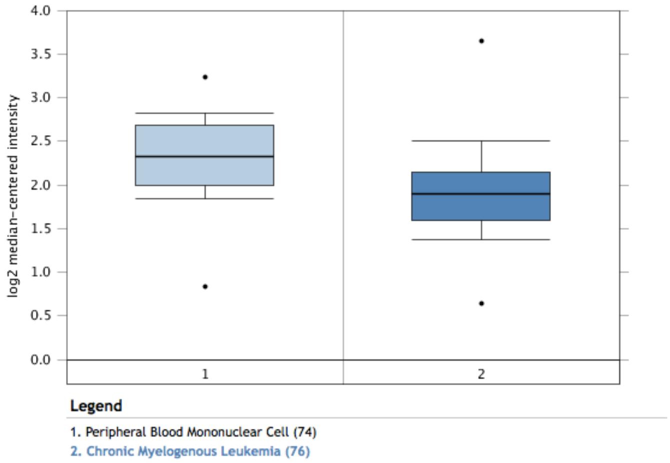

B

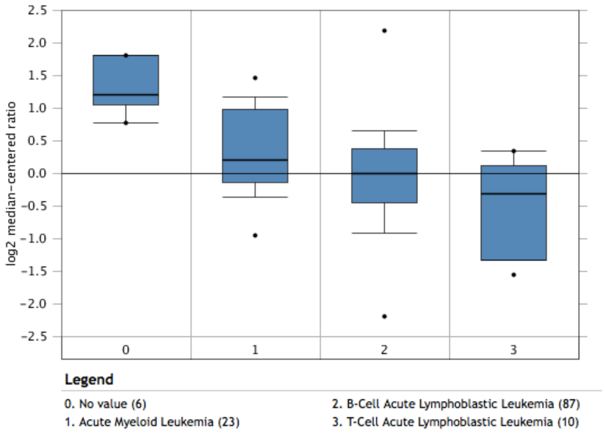

Supplementary Fig. 1 (A) Oncomine was used to analyze the USP15 expression level in patients with CML and normal cells in TCGA. (B) Oncomine was used to analyze the USP15 expression level in patients with AL and normal cells in TCGA.

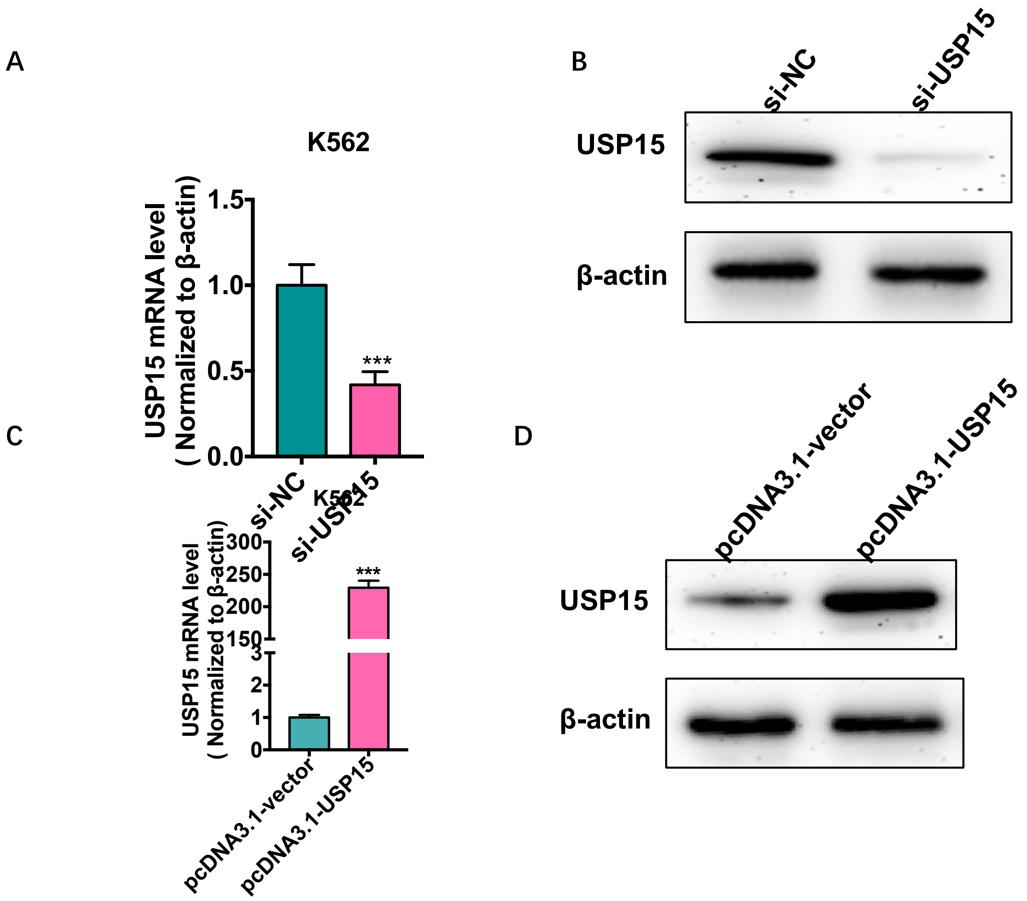

Supplementary Fig. 2 (A) K562 cells were transfected with si-USP15 or si-NC. USP-15 mRNA level was test by qRT-PCR. ***P< 0.001 vs. si-NC. (B) K562 cells were transfected with si-USP15 or si-NC. Protein level of USP-15 was test by Western blot analysis. (C).K562 cells were transfected with USP15 overexpression vector pcDNA3.1-USP15 or empty vector. USP-15 mRNA level was test by qRT-PCR. ***P< 0.001 vs. pcDNA3.1 vector. (D).K562 cells were transfected with USP15 overexpression vector pcDNA3.1-USP15 or empty vector. Protein level of USP-15 was test by Western blot analysis.

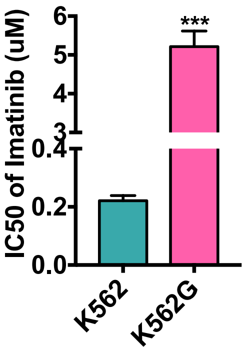

Supplementary Fig. 3 K562 and K562G cells were treated with Imatinib in different concentrations for 48 h. Imatinib IC50 was evaluated by CCK-8 assay. ***P<0.001 vs K562 cell.

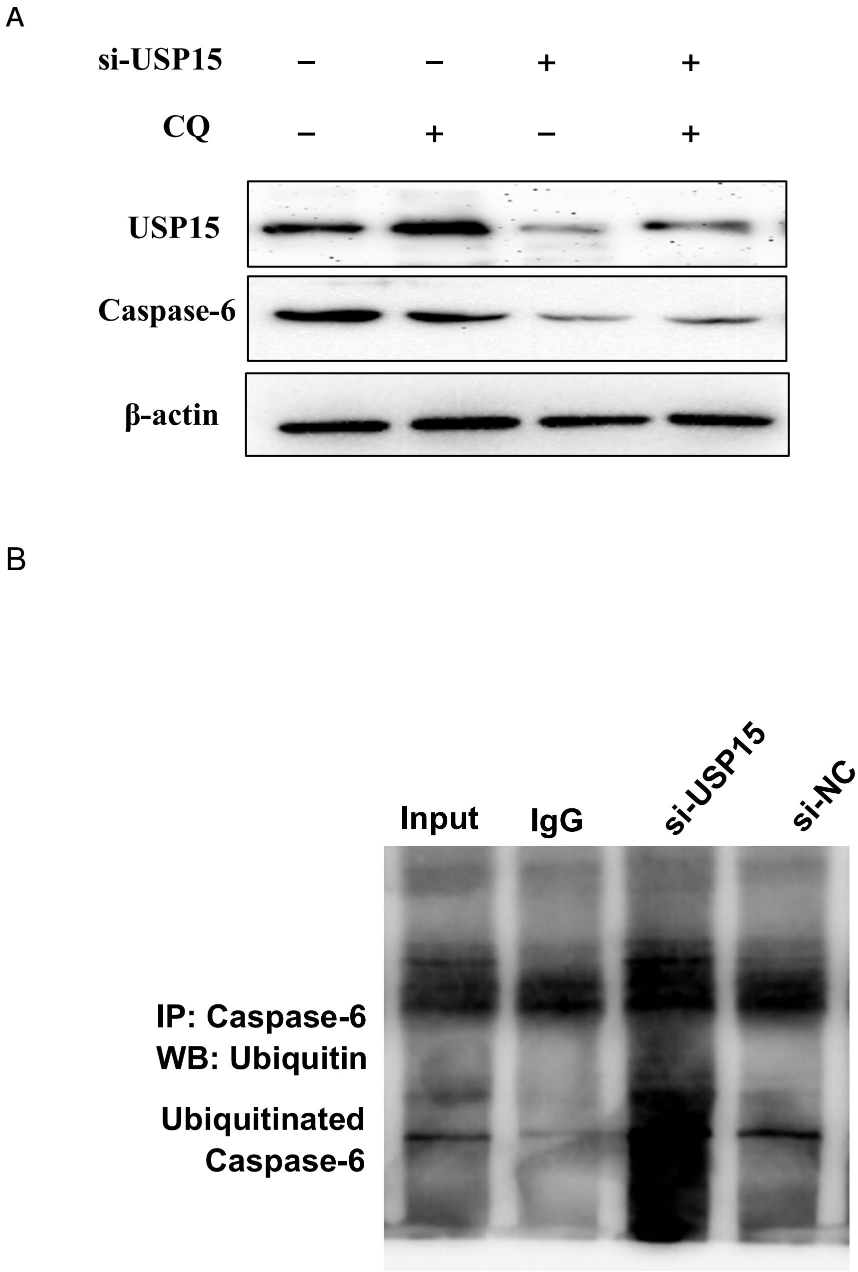

Supplementary Fig. 4 (A) K562 cells were treated with CQ for 12h or not and then transfected with si-NC or si-USP15 for 24 h then. USP15 and Caspase-6 protein level was detected by Western blot analysis. (B) K562 cells were then transfected with si-NC or si-USP15 for 24 h then MG132 for 6 h. Co-immunoprecipitation (CoIP) assays were performed to detect the interaction between Ubiqutin and Caspase-6.

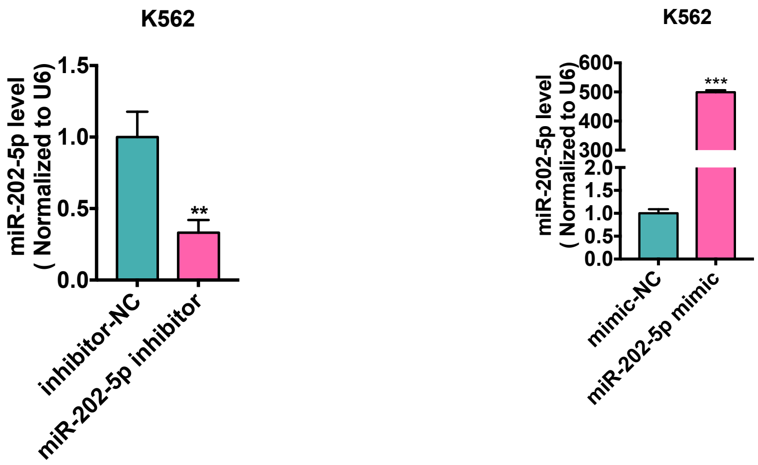

Supplementary Fig. 5 (A) K562 cells were transfected with pre-miR-202-5p inhibitor or inhibitor-NC. Pre-miR-202-5p expression level was detected by qRT-PCR. ***P*<0.01 vs. inihibitor-NC. (B) K562 cells were transfected with miR-202-5p mimic or mimic-NC. miR-202-5p expression level was detected by qRT-PCR. ** *P*<0.01 vs. mimic-NC.

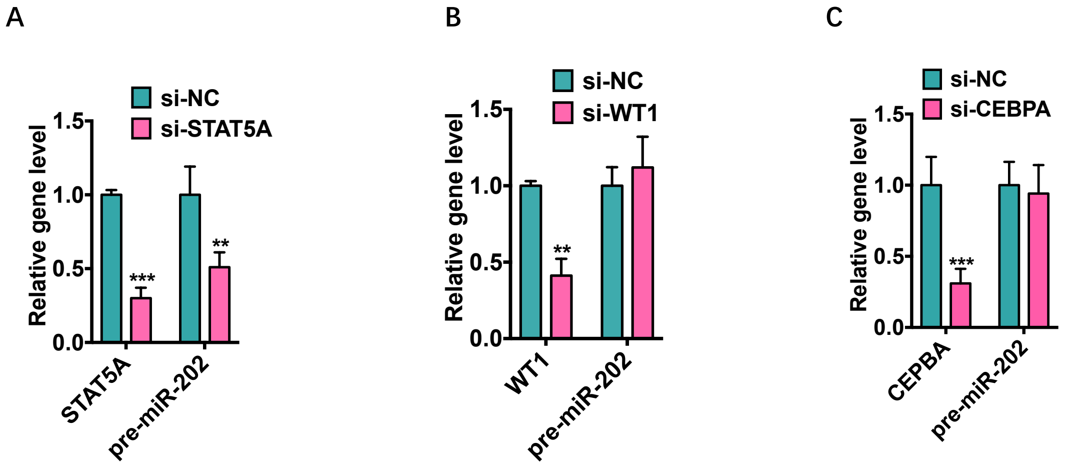

Supplementary Fig. 6 (A) K562 cells were transfected with si-STAT5A or si-NC . miR-202-5p and STAT5A expression levels were detected by qRT-PCR. ** *P*<0.01, *** *P*<0.001 vs. si-NC. (B) K562 cells were transfected with si-WT1 or si-NC . miR-202-5p and WT1 expression levels were detected by qRT-PCR. ** *P*<0.01 vs. si-NC. (C) K562 cells were transfected with si-CEBPA or si-NC . miR-202-5p and CEBPA expression levels were detected by qRT-PCR. ** **P*<0.001, vs. si-NC.

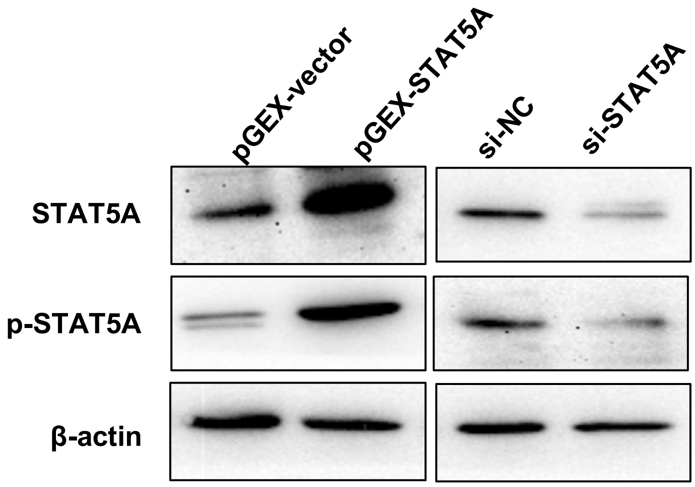

Supplementary Fig. 7 K562 cells were transfected with pGEX-STAT5, pGEX-vector, si-STAT5A and si-NC, respectively. Protein levels of STAT5A and p-STAT5A was detected by Western blot analysis.

Supplementary Table. 1 Interaction Proteins with USP15 in CoIP-MS analysis

Score	Mass	Name
655	11360	Histone H4
521	42052	Actin, cytoplasmic 1
82	134145	Serine/threonine-protein kinase Nek10
360	58415	Alpha-amylase 1
238	83554	Heat shock protein HSP 90-beta
226	18386	Selenoprotein F
176	113602	Ubiquitin carboxyl-terminal hydrolase
136	2220	T-cell receptor alpha joining 56 (Fragment)
126	10463	Polyubiquitin-B (Fragment)
122	30759	Apolipoprotein A-I
105	73920	Stress-70 protein
95	58606	Tubulin alpha chain
85	229827	Myosin-10
79	11391	Dermcidin
67	33859	Caspase-6
57	49680	Vimentin
56	13291	Protein S100-A9
56	48195	Elongation factor 1-alpha 1
56	10885	Protein S100-A8
55	38366	Proto-oncogene serine/threonine-protein kinase mos
55	19135	Peroxiredoxin-1 (Fragment)
53	26683	Protein AMBP OS=Homo sapiens
53	98164	Inactive rhomboid protein 2
52	59828	ATP synthase subunit alpha, mitochondrial
51	38816	Transcription factor AP-4

Supplementary Table. 2 Clinical Characteristics

Characteristics	CML-CP (n=30)	Donors (n=30)	CML-AML (n=4)	CML-ALL (n=2)
**Age(years),median(range)**	42(29-71)	42(29-71)	51(44-62)	56(52-60)
**Male/Female,(n/n)**	18/12	18/12	1/3	1/1
**WBCs,×10^9/L, median(range)**	249(39-405)	6.1(4.9-9.3)	29.1(9.2-30.5)	36.6(30.4-42.8)
**Hb level (g/L)**	99(81-146)	139(121-155)	67(52-77)	61(51-71)
**PLT , ×10^9/L, median(range)**	464(94-797)	221(104-312)	35(11-66)	22(14-30)
