# Supplementary material for: De-regulated STAT5A/miR-202-5p/USP15/Caspase-6 regulatory axis suppresses CML cell apoptosis and contributes to Imatinib resistance
Source: J Exp Clin Cancer Res. 2020 Jan 17;39:17. doi: 10.1186/s13046-019-1502-7 (PMC6969434; doi:10.1186/s13046-019-1502-7)
Supplement: Supplementary file 2 — Additional file 2. Supplemental Experimental Procedures. [file 13046_2019_1502_MOESM2_ESM.docx]

**Supplementary information**

**Cell transfection**

Cells were transfected with Lipofectamine 2000 (Invitrogen) according to the manufacturer’s protocols. The miR-202-5p mimic/inhibitor, si-STAT5A, si-USP15, si-Caspase-6, si-WT1 and si-CEBPA and their respective negative controls were purchased from GenePharma Co.Ltd (Shanghai, China). The overexpression plasmids of USP15 and STAT5A and luciferase assay plasmids were purchased from GENEWIZ Company (Suzhou, China).

**Cell viability assay**

Cell viability was tested by Cell Counting Kit-8 (CCK-8, Beibo, Beijing, China) following manufacturer’s protocols. Briefly, cells were seeded in 96-well plates and treated with Imatinib or Pimozide at the different concentrations for 48 h, respectively. Finally, the absorbance was measured at 450 nm using a microplate reader (Thermo Fisher USA).

**Analyses of apoptosis**

Cells grown in 6-well plates were treated with Imatinib or Pimozide for 48 h, respectively. For drug combination studies, cells were treated with Pimozide and Imatinib for 48 h. The Annexin V-FITC/PI apoptosis detection kit (BD Biosciences, USA) was used to detect cell apoptosis following the manufacturer’s instructions. The data analysis was performed using BD FACS Diva software (BD, USA).

**Xenograft animal model**

All animal studies were approved by the Institutional Animal Care Committee of Hebei Medical University. 6 weeks' old BALB/c nude mice were purchased from Vital River Laboratory Animal Technology Co., Ltd (Beijing, China). To test the effect of miR-202-5p in CML, 1 × 10^7^ LV-anti-miR-202-5p or LV-miR-anti-NC-infected K562 cells were resuspended in 50 μL PBS mixed 50 μL Matrigel (356234. BD, MA, USA) (each group n=6); this suspension was injected subcutaneously into the left dorsal flanks. To test the effect of pimozide in CML, 1 × 10^7^ K562 was injected subcutaneously into the left dorsal flanks. Following a week inoculation period, the tumor-bearing mice were randomly divided into pimozide treatment group and control groups (each group n=6). In treatment group, pimozide was administered to mice intraperitoneally five times/week for 2 weeks. And the control group was injected with the saline. The volume of xenograft was measured twice a week. At the end of the experiment (28 days), the mice were euthanized by Carbon dioxide asphyxiation. The tumor tissues were stored in liquid nitrogen or fixed in 4% paraformaldehyde immediately and stored at −80 °C until further use.

**RNA extraction and quantitative real-time PCR**

RNA Purification Kit (RNAeasy Mini Elute kit, QIAGEN) were used to prepare total RNAs form tissues and culture cells according to the manufacturer's protocol. The concentration and purity of total RNA were measured by using Nanodrop spectrophotometer (Thermo Fisher). For microRNA analysis, the miScripIIRT kit (QIAGEN GmbH, D-40724 Hilden, GERMANY) was used for reverse transcription, and the miScript SYBR® Green PCR kit was used for qRT-PCR with specific primers for microRNAs using the following primer: miR-202-5p: GGCCTTCCTATGCATATACTTCTTTG; U6:GTGCTCGCTTCGGCAGCACATATAC. RNU6b (U6) was used as internal control. For mRNA analysis, total cellular RNA was reverse-transcribed to first strand cDNA with M-MLV First Strand Kit (Life Technologies). And Platinum SYBR Green qPCR Super Mix UDG Kit (Invitrogen) was used for the qRT-PCR of mRNAs using the following primers:USP15-F:5’-ATAGTGTCCTCCTCCTCCCATCCC -3’, USP15-R5’-TTGTAAAGAACATCAGCAAGCC-3’: CEBPA-F:TAGTGGAAAACCAGCCTCCC, CEBPA-R:GTGTGACCGCAACGTAGGAG; STAT5A-F:CTGAAGATCAAGCTGGGGCACTAC, STAT5A-R:CTCATTCTCTGTGTCCTGCGTGAC;WT1-F:CGATAACCACACAACGCCCATC, WT1-R:GTGCTTCCTGCTGTGCATCTGTAA; pre-miR-202-F:CGCCTCAGAGCCGCCCGCC; pre-miR-202-R:CGCTGGGGAGGACCCGACCG; β-actin-F: GAGCTACGAGCTGCCTGAC; β-actin-R:5’-GGTAGTTTCGTGGATGCCACAG-3’. The mRNA expression was normalized to that of β-actin calculated using the 2−ΔΔCt formula.

**Western blot analysis**

The protein was extracted from the cultured cells and frozen tissue samples with RIPA lysis buffer. Equal amounts of protein were run on 10% SDS-PAGE, and electro-transferred to a polyvinylidene fluoride (PVDF) membranes (Millipore). After blocking in 5% nonfat milk, the membranes were incubated with specific primary antibodies as follows: anti-USP15 (1:1000, 14354-1-AP, Proteintech), anti-caspase-6 (1:500, 10198-1-AP, Proteintech), anti-STAT5A (1:2000, 94205, CST), anti-p-STAT5A (1:2000, 9351, CST) or anti-β-actin (1:5000, ab6276 Abcam). The proteins were visualized with Immobilon ECL (Millipore). FusionCapt Advance Fx5 software (Vilber Lourmat) was used to capture the images.

***In situ* hybridization**

*In situ* hybridization (FISH) was performed following manufacturer’s instructions of miRCURY LNATM microRNA ISH Optimization Kit (Exiqon) as described previously[1]. In brief, all the cell smears were deparaffinized and rehydrated for fluorescence *in situ* hybridization. Hybridization was performed using fluorescence-labeled miR-202-5p probes with hybridization buffer (Exiqon) by incubation at 55 °C for 1 h. Then the smears were stringently washed with SSC buffer and PBS. Leica microscope (Leica DM6000B, Switzerland) was used to acquire the images. And all images were digitized with a software of LAS V.4.4 (Leica).

**Vector construction and luciferase reporter assay**

Luciferase assay analysis was performed as described previously[2]. For miRNA luciferase reporter assay, K562 cells were seeded into a 24-well plate, miR-202-5p mimic (or mimic-NC) was co-transfected with USP15 reporter construct (wild-type or mutant) or the empty vector. For promotor luciferase reporter assay, K562 cells were seeded into a 24-well plate, pre-miR-202 reporter construct or the empty reporter vector was co-transfected with pGEX-STAT5 and pRL-TK, or co-transfected with pGEX-vector and pRL-TK . After 24 h after transfection, the cells were harvested in lysis buffer. Dual-Glo Luciferase Assay System (Promega, Madison, WI) was used to detect luciferase activity according to the manufacturer’s protocols. Firefly luciferase activity was measured and normalized against the Renilla luciferase activity.

**Immunofluorescence staining**

4% formaldehyde was used to fix the cells, and the cell smears were pre-incubated with 10% normal goat serum (710,027, KPL, USA). Then the smears were incubated with primary antibody anti-USP15(sc-100629, Santa) and anti-caspase-6 (10198-1-AP, Proteintech) at 37℃ for 1 h and then was incubated in streptavidin (HRP)-biotin labeled secondary antibody. Images were acquired using a Leica microscope (Leica DM6000B, Switzerland) and digitized with LAS V.4.4 (Leica).

**Co-immunoprecipitation assay**

Co-immunoprecipitation analysis was performed as described previously[3]. Briefly, cultured cells were lysed by RIPA and then lysates were immunoprecipitated with anti-caspase-6 or anti-USP15 for 1 hour at 4 °C. Protein A-agarose were added to the lysates for incubating overnight. Next day, Protein A-agarose-antigen-antibody complexes were collected by centrifugation at 12,000 g for 2 min at 4 °C and immunoprecipitation-HAT buffer was used to washed complexes for 5 times. Western blot was used to detect the bound proteins. Differentially interacted proteins of USP15 were identified by using Mass Spectrometry on the Ultrafle Xtreme LC-MS/MS mass spectrometer at Kangchen in Shanghai, China.

**Chromatin immunoprecipitation (ChIP) assay**

The chromatin immunoprecipitation (ChIP) assay was using the was performed as described previously[4]. In brief, K562 cells were treated with 1% formaldehyde to cross-link proteins with DNA. The cross-linked chromatin was then prepared and sonicated to an average size of 400–600 bp. The samples were diluted 10-fold and then precleared with protein A-agarose/salmon sperm DNA for 30 min at 4 °C. The DNA fragments were immunoprecipitated overnight at 4 °C with anti-STAT5A or anti-IgG (as negative control) antibodies. After cross-linking reversal, STAT5A on pre-miR-202 promotor was examined. Results were determined by qRT-PCR with the following primers: STAT5-chip-F1: GGCTCAGAGAGTTCTCCACCGCTC, STAT5-chip-R1: CGGCTCTTCAGCACTGGGAGGTG; STAT5-chip-F2: GTCCGCCTGATGCCGTGAATC, STAT5-chip-R2: CCAGGGAAGGGGCCGACTCTC; STAT5-chip-F3: CGAGGACATGGTCACTGAAGAGG; STAT5-chip-R3: CCAGGCAGGCAGCAGAACTCC.

**Highlight sequence of miRNA**

Highlight sequence of miRNA was sequenced using the Illumina Hiseq 2000 platform at Anoroad, Beijing, China. Library preparation, cluster generation and sequencing by synthesis were performed by using SE50 sequencing strategy according to manufacturer’s protocol[5]. Differential analysis using DEsqe software and following the principle: pad j<0.05 and log_2_(Fold_change)>1 or <-1.

**RNA synthesis and Biotin pull-down**

RNA was synthesized by in vitro transcription as previously described[6]. PCR primers including T7 promoter sequences of USP5 3' UTR were designed and amplified from K562 cell line genomic DNA. Biotin-labeled RNA was synthesized by in vitro transcription using MEGAscript T7 transcription kit (Ambion, AM1334) and adding Biotin-16-UTP (Ambion, AM8452) in a 1:25 ratio with the transcription kit. miRNeasy Mini Kit (217004; Qiagen) was purified for transcribe RNA according to the manufacturer's instructions. Biotin pull-down was carried out to detected USP15 3' UTR and microRNAs interaction as previously described[6] .

**References:**

1. Yang Z, Zheng B, Zhang Y, He M, Zhang XH, Ma D, Zhang RN, Wu XL, Wen JK: **miR-155-dependent regulation of mammalian sterile 20-like kinase 2 (MST2) coordinates inflammation, oxidative stress and proliferation in vascular smooth muscle cells**. *Bba-Mol Basis Dis* 2015, **1852**(7):1477-1489.

2. Yang Z, Chen JS, Wen JK, Gao HT, Zheng B, Qu CB, Liu KL, Zhang ML, Gu JF, Li JD *et al*: **Silencing of miR-193a-5p increases the chemosensitivity of prostate cancer cells to docetaxel**. *J Exp Clin Cancer Res* 2017, **36**(1):178.

3. Zhang XH, Zheng B, Yang Z, He M, Yue LY, Zhang RN, Zhang M, Zhang W, Zhang X, Wen JK: **TMEM16A and myocardin form a positive feedback loop that is disrupted by KLF5 during Ang II-induced vascular remodeling**. *Hypertension* 2015, **66**(2):412-421.

4. Yang Z, Qu CB, Zhang Y, Zhang WF, Wang DD, Gao CC, Ma L, Chen JS, Liu KL, Zheng B *et al*: **Dysregulation of p53-RBM25-mediated circAMOTL1L biogenesis contributes to prostate cancer progression through the circAMOTL1L-miR-193a-5p-Pcdha pathway**. *Oncogene* 2019, **38**(14):2516-2532.

5. Schwientek P, Wendler S, Neshat A, Eirich C, Ruckert C, Klein A, Wehmeier UF, Kalinowski J, Stoye J, Puhler A: **Comparative RNA-sequencing of the acarbose producer Actinoplanes sp. SE50/110 cultivated in different growth media**. *J Biotechnol* 2013, **167**(2):166-177.

6. Sun Y, Yang Z, Zheng B, Zhang XH, Zhang ML, Zhao XS, Zhao HY, Suzuki T, Wen JK: **A Novel Regulatory Mechanism of Smooth Muscle alpha-Actin Expression by NRG-1/circACTA2/miR-548f-5p Axis**. *Circ Res* 2017, **121**(6):628-635.
